# The energy budget in C_4_ photosynthesis: insights from a cell‐type‐specific electron transport model

**DOI:** 10.1111/nph.15051

**Published:** 2018-03-09

**Authors:** Xinyou Yin, Paul C. Struik

**Affiliations:** ^1^ Department of Plant Sciences Centre for Crop Systems Analysis Wageningen University & Research PO Box 430 6700 AK Wageningen the Netherlands

**Keywords:** bioenergetics, C_4_ modelling, C_4_ photosynthesis, cell type, cyclic electron transport, energy balance, mixed decarboxylation, quantum yield

## Abstract

- Extra ATP required in C_4_ photosynthesis for the CO_2_‐concentrating mechanism probably comes from cyclic electron transport (CET). As metabolic ATP : NADPH requirements in mesophyll (M) and bundle‐sheath (BS) cells differ among C_4_ subtypes, the subtypes may differ in the extent to which CET operates in these cells.
- We present an analytical model for cell‐type‐specific CET and linear electron transport. Modelled NADPH and ATP production were compared with requirements.
- For malic‐enzyme (ME) subtypes, *c*. 50% of electron flux is CET, occurring predominantly in BS cells for standard NADP‐ME species, but in a ratio of *c*. 6 : 4 in BS : M cells for NAD‐ME species. Some C_4_ acids follow a secondary decarboxylation route, which is obligatory, in the form of ‘aspartate‐malate’, for the NADP‐ME subtype, but facultative, in the form of phosphoenolpyruvate‐carboxykinase (PEP‐CK), for the NAD‐ME subtype. The percentage for secondary decarboxylation is *c*. 25% and that for 3‐phosphoglycerate reduction in BS cells is *c*. 40%; but these values vary with species. The ‘pure’ PEP‐CK type is unrealistic because its is impossible to fulfil ATP : NADPH requirements in BS cells.
- The standard PEP‐CK subtype requires negligible CET, and thus has the highest intrinsic quantum yields and deserves further studies in the context of improving canopy productivity.

## Introduction

The CO_2_‐concentrating mechanism (CCM) in C_4_ leaves relies on the coordinated functioning of two distinct photosynthetic cell types, mesophyll (M) cells and bundle‐sheath (BS) cells (Hatch, 1987; Kromdijk *et al*., 2010). CO_2_ from the ambient air diffuses first to M cells where CO_2_ is converted into HCO_3_^−^ and then fixed by phospho*enol*pyruvate carboxylase (PEPc) to produce oxaloacetate (OAA). Quickly OAA is either reduced to malate or converted into aspartate. These C_4_ acids diffuse to BS cells where they are decarboxylated to deliver CO_2_ to Rubisco to start the Calvin cycle or the C_3_ cycle (Hatch, 1987). As rates of carboxylation by PEPc are faster than those of carboxylation by Rubisco (Sage *et al*., 1987), the CO_2_ content in BS cells becomes high, thereby suppressing photorespiration, despite some leakiness (a fraction of decarboxylated CO_2_ in the BS cells that leaks back to M cells).

Operation of the CCM involves the regeneration of PEP in the C_4_ cycle, which requires ATP (Hatch, 1987; von Caemmerer & Furbank, 1999), in addition to ATP required by the C_3_ cycle. Although the CO_2_ fixation by Rubisco occurs exclusively in BS cells, the enzymes of the reductive phase of the C_3_ cycle are found in both M and BS chloroplasts, suggesting that 3‐phosphoglycerate (3‐PGA) is reduced in both cell types (Kanai & Edwards, 1999; Majeran *et al*., 2005; Friso *et al*., 2010). In addition, the full C_4_ cycle involves both types of cells (Supporting Information Fig. S1), and the amount and location of the ATP required for PEP regeneration depend on the C_4_ subtypes (Hatch, 1987; Kanai & Edwards, 1999). The subtypes were traditionally classified according to the enzymes that decarboxylate C_4_ acids in BS compartments: NADP‐malic enzyme (ME) in chloroplasts, NAD‐ME in mitochondria and PEP carboxykinase (CK) in the cytosol (Hatch, 1987; von Caemmerer & Furbank, 2003).

In the NADP‐ME subtype, OAA is reduced to malate by NADP‐malate dehydrogenase (NADP‐MDH) using NADPH in M chloroplasts (Fig. S1). The malate then moves to BS chloroplasts where malate is decarboxylated by NADP‐ME, releasing pyruvate, which is transported back to M chloroplasts and regenerated to PEP by pyruvate, phosphate dikinase (Kanai & Edwards, 1999; Bräutigam *et al*., 2014). Decarboxylation of malate also releases NADPH in BS chloroplasts. Thus, if half of the 3‐PGA reduction occurs in M chloroplasts, 1 mol NADPH for the reduction of 1 mol 3‐PGA in BS chloroplasts can be fully met by the decarboxylation of 1 mol malate. So, BS photosynthetic electron transport would not need to produce NADPH but only to produce ATP (Hatch, 1987; Kanai & Edwards, 1999). The BS chloroplasts of NADP‐ME species indeed have little photosystem II (PSII) (Woo *et al*., 1970; Gutierrez *et al*., 1974), which is indispensable for the linear electron transport (LET) to produce NADPH. So, for the simplest case assuming no leakiness, 2 mol ATP (1 mol for the reduction of 1 mol 3‐PGA and 1 mol for the regeneration of 1 mol RuBP) in BS cells, and 2 mol NADPH (1 mol for the reduction of 1 mol 3‐PGA and 1 mol for the reduction of 1 mol OAA to malate) and 3 mol ATP (2 mol for the regeneration of 1 mol PEP and 1 mol for the reduction of 1 mol 3‐PGA) in M cells should be supplied by photosynthetic electron transport per mol CO_2_ fixed (Hatch, 1987; Kanai & Edwards, 1999).

In the NAD‐ME subtype, OAA is converted into aspartate in the M cytosol by aspartate aminotransferase for transport to BS cells. Aspartate is converted to OAA again in the BS cytosol by aspartate aminotransferase. In BS cells, the mitochondria provide NADH for reducing OAA to malate, and are also the site of decarboxylation (Fig. S1). If the reduction of 3‐PGA equally occurs in M and BS chloroplasts (Hatch, 1987), 1 mol NADPH and 2 mol ATP (1 mol for 3‐PGA reduction and 1 mol for RuBP regeneration) are required in BS cells, and 1 mol NADPH and 3 mol ATP (1 mol for 3‐PGA reduction and 2 mol for PEP regeneration) are required in M cells, per mol CO_2_ fixed.

In the PEP‐CK subtype, a portion of OAA is reduced to malate (using NADPH from M chloroplasts) and its remaining portion travels, via aspartate, to BS cytosol, and malate decarboxylation occurs in mitochondria of BS cells by NAD‐ME simultaneously with the direct decarboxylation of OAA in BS cytosol by PEP‐CK (Fig. S1). One mol ATP is required per mol OAA directly decarboxylated by PEP‐CK (Kanai & Edwards, 1999); this extra ATP is exclusively produced by NADH oxidation in the respiratory chain of BS mitochondria associated with malate decarboxylation (Hatch, 1987; Burnell & Hatch, 1988). Therefore, this PEP‐CK subtype requires additional NADPH to operate, which depends on the stoichiometry of ATP production per oxidation of NADH. If this ATP : NADH ratio (*n*) is 2.5 (Hinkle *et al*., 1991; Kanai & Edwards, 1999), then 0.286 mol extra NADPH is required [solved for *a* from *na *= 1(1‐ *a*)] per mol CO_2_ fixed. In this case, 0.572 mol ATP is required for PEP regeneration in M chloroplasts to drive NAD‐ME‐dependent decarboxylation. As a result, the minimum energy requirements for the PEP‐CK subtype are 3.572 mol ATP and 2.286 mol NADPH per mol CO_2_ assimilated (Kanai & Edwards, 1999). If *n* is 3.0 (Ferguson, 1986; von Caemmerer & Furbank, 1999), the minimum requirements will be 3.50 mol ATP and 2.25 mol NADPH. Assuming that 3‐PGA is reduced equally in M and BS chloroplasts, the energy requirements are 1.572 (or 1.50) mol ATP and 1.286 (or 1.25) mol NADPH in M cells and 2 mol ATP and 1 mol NADPH in BS cells per mol CO_2_ fixed. So, in terms of ATP requirement from the chloroplast, this PEP‐CK subtype is the most efficient C_4_ type, despite costing additional NADPH.

Whilst this third type is called the PEP‐CK subtype, it is actually a mixture of NAD‐ME and PEP‐CK. A ‘pure’ PEP‐CK type appears to be a theoretical type, not appearing in nature (von Caemmerer & Furbank, 2016). In fact, there has been renewed discussions on engaging a mixed decarboxylating pathway (Furbank, 2011; Stitt & Zhu, 2014). The PEP‐CK mechanism is probably present in NADP‐ME species such as maize which transport some aspartate to the BS (Pick *et al*., 2011; Sommer *et al*., 2012; Koteyeva *et al*., 2015). The NADP‐ME species *Flaveria bidentis* partially recruits an NAD‐ME‐like pathway (Meister *et al*., 1996). *Cleome gynandra* and *Eragrostis nutans* were identified by Sommer *et al*. (2012) and Koteyeva *et al*. (2015), respectively, as NAD‐ME type having substantial amounts of PEP‐CK. Numerical modelling studies suggest advantages of the mixed decarboxylation in accommodating to varying light conditions (Bellasio & Griffiths, 2014; Wang *et al*., 2014).

Regardless of the C_4_ subtypes or the mixed types, there needs to be a balance in each cell type between supply and demand for ATP/NADPH. This balance is important under steady‐state and dynamic environmental conditions because any mismatch will rapidly (within seconds) inhibit photosynthesis as chloroplasts have limited pools of NADPH and ATP (Kramer & Evans, 2011). Whole‐leaf model analysis of Yin & Struik (2012) by matching quantum yield of CO_2_ assimilation (Φ_CO2_) with that of PSII photochemistry under limiting light (Φ_2LL_) showed that a high level of cyclic electron transport around PSI (CET) was required, and CET accounted for *c*. 45% of total PSI electron flux in NADP‐ME and NAD‐ME subtypes, providing extra ATP required for CCM. Recent literature (e.g. Nakamura *et al*., 2013; Ishikawa *et al*., 2016; Munekage & Taniguchi, 2016) supported the central role of CET for C_4_ photosynthesis. Furthermore, as introduced earlier, the ATP : NADPH ratio in NADP‐ME species is 2 : 0 and 3 : 2, but the ratio in NAD‐ME species is 2 : 1 and 3 : 1, in BS and M cells, respectively, for the simplest case (Fig. S1). This suggests that the activity of CET should be substantially higher in BS cells of NADP‐ME species than that of NAD‐ME species. Indirect evidence linking CET to the extra ATP requirement is that PSI : PSII ratios in the BS cells of NADP‐ME species are higher than those of NAD‐ME species (Hatch, 1987; Ghannoum *et al*., 2005; Iermak *et al*., 2016).

Although higher CET satisfies the local high ATP requirement, high CET reduces whole‐leaf Φ_CO2_ (Yin *et al*., 2004) and is the major constraint to canopy productivity of C_4_ crops that belong to the NADP‐ME subtype (Yin & Struik, 2017). Any model quantifying the trade‐off between cell‐type‐specific energy requirements and whole‐leaf Φ_CO2_ needs to consider to what extent CET operates in each cell type. Current models on C_4_ energy budgets either do not incorporate CET explicitly (e.g. Wang *et al*., 2014) or are tailored to the NADP‐ME subtype, where CET is assumed to occur exclusively in BS cells and LET exclusively in M cells (Bellasio & Griffiths, 2014; Bellasio & Lundgren, 2016).

The objective of this study was to quantify the energy budgets in M and BS cells of various C_4_ subtypes. Specifically, we aimed to analyse (1) whether cell‐type‐specific ATP and NADPH demands can be met by cell‐type‐specific electron transport, and (2) if not, what strategies various C_4_ subtypes would need to maintain the supply–demand balance of ATP/NADPH in each cell type. To avoid any confounding effect of processes other than cellular energetics, we focused on steady‐state light conditions, under which photosynthesis is limited by electron transport. This also allowed us to calculate whole‐leaf Φ_CO2_ by developing an analytical model for both CET and LET in both M and BS cells, without relying on computationally expensive numerical simulation.

## Materials and Methods

**Figure 1:**
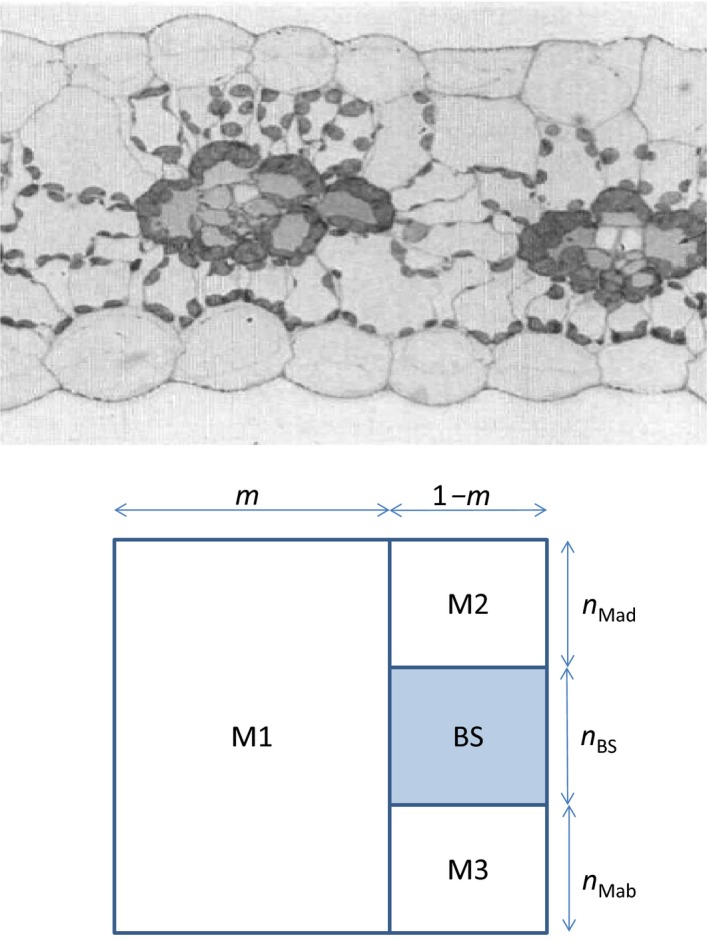
Upper panel: two units of interveinal distance of a maize leaf (redrawn from Evans & von Caemmerer, 1996; with permission); lower panel: schematic representation of bundle‐sheath (BS) and mesophyll (M) sections of one unit of interveinal distance. The BS section is shaded, with relative height *n*_BS_. The M part has three sections: interveinal M (M1), the adaxial side above BS (M2) and the abaxial side below BS (M3). The relative heights of M2 and M3 are denoted as *n*_Mad_ and *n*_Mab_, respectively; thus, the relative height of M1 is (*n*_Mad_ + *n*_BS_ + *n*_Mab_), which together makes one full relative height. In the model, it is assumed for simplicity that *n*_Mad_ = *n*_Mab_ = (1‐ *n*_BS_)/2, based on many published images of C_4_ leaves (e.g. Ghannoum *et al*., 2005). The fraction of one unit interveinal distance for M1 is *m*; the remaining fraction, 1‐ *m*, is then the vein width. So, areas of BS, M1, M2 and M3 sections can be easily calculated from *m* and *n*_BS_. The total Chl content in M cells can be partitioned among the three M sections according to their areas relative to the total M area. In a real leaf, M1 is commonly divided into two pieces that are placed on both left and right sides of the M2–BS–M3 vein area (Bellasio & Lundgren, 2016), and the two subsections of M1 and the M2–BS–M3 section together form the interveinal distance. Here, the two subsections are combined into a single M1 for mathematical simplicity, but this has no influence on our modelling results.

Our model for NADPH and ATP production, based on a simple cell‐type structure (Fig. 1), is fully described in Methods S1. For the reasons described therein, we first model the basic situation in which only CO_2_ fixation is considered, and then alternative electron and ATP sinks are introduced. For either situation, Φ_CO2_ was modelled to be co‐limited by NADPH and ATP production, given the importance of achieving metabolically required ATP : NADPH ratios (Kramer & Evans, 2011). The approach was applied to the four C_4_ species studied by Ghannoum *et al*. (2005), with available data as model input. Model source codes are given in Methods S2.

## Results and Discussion

### General performance of the basic model

**Figure 2:**
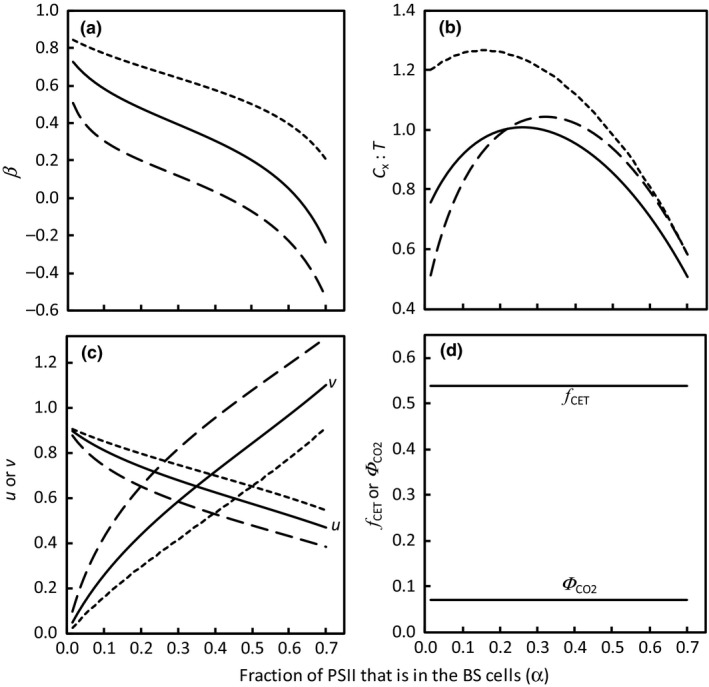
Simulated values of (a) fraction of photosystem I (PSI) used for cyclic electron transport (CET) that is in BS cells (β), (b) the ratio of PSI used for CET to total PSII (*C*_*x*_ : *T*), (c) fraction of mesophyll (M) cell‐absorbed light that is used for linear electron transport (LET) (*u*, descending curves) and fraction of bundle sheath (BS) cell‐absorbed light for LET (*v*, ascending curves), and (d) quantum yield (Φ_CO2_) and fraction of whole‐leaf CET (*f*_CET_), as a function of parameter α (the fraction of total PSII that is in the BS cells), using three values of *f*_bsPSI_, the fraction of PSI in BS cells (0.20, 0.35 and 0.50 for long‐dashed, solid and short‐dashed curves, respectively). Dashed curves are invisible in (d) as the same Φ_CO2_ and *f*_CET_ were predicted with different values of *f*_bsPSI_.

For the simulation using the basic model where only CO_2_ fixation consumes NADPH and ATP (section 1 of Methods S1), we used the parameter values in Table 1. Some of these values may be uncertain and differ between subtypes or individual species. Modelled results for the fraction of PSI used for CET that is in BS cells (β, Fig. 2a) and the ratio of PSI used for CET to total PSII (*C*_x_ : *T*, Fig. 2b) are shown in Fig. 2 as a function of α, the fraction of PSII in BS cells. The *C*_x_ : *T* ratio showed a complex relationship with α, depending on the fraction of PSI in BS cells (*f*_bsPSI_) (Fig. 2b).

**Table 1:** Indicative values of model input parameters

Symbol	Definition	Unit	Value	Source
*m*	Fraction of one unit interveinal distance for the M1 section in Fig. 1	–	0.55	Bellasio & Lundgren (2016)^a^
*n*_BS_	Fraction of one unit depth for the bundle‐sheath (BS) section in Fig. 1	–	0.6	Derived from Griffiths *et al*. (2013)^b^
Φ_2LL_	Efficiency of PSII electron transport	mol mol^−1^	0.8^c^	Genty *et al*. (1989)
Φ_2LL_/Φ_1LL_	Ratio of PSII : PSI electron transport efficiency	–	0.85	Genty & Harbinson (1996)
[CHL]	Leaf Chl content	µmol Chl m^−2^	475	Ghannoum *et al*. (2005)
*f*_bsCHL_	Fraction of [CHL] in BS cells	–	0.33	Ghannoum *et al*. (2005)
*f*_bsPSI_	Fraction of PSI in BS cells	–	0.35	Ghannoum *et al*. (2005)
*k*	Light extinction coefficient	m^2^ (µmol Chl)^−1^	0.005	Fitted to agree with the whole‐leaf absorptance^d^
*H*_LET_	Proton (H^+^) : e^−^ ratio of LET	mol H^+^ (mol e^−^)^−1^	3	Allen (2003)
*H*_CET_	Proton (H^+^) : e^−^ ratio of CET	mol H^+^ (mol e^−^)^−1^	2	Yin & Struik (2012)
*h*	H^+^ : ATP ratio	mol H^+^ (mol ATP)^−1^	4	Yin & Struik (2012)
ϕ	Leakiness	–	0.16^e^	Yin & Struik (2012)
φ	Extra chloroplastic ATP required per C_4_ cycle	mol ATP (mol CO_2_)^−1^	2^f^	von Caemmerer & Furbank (1999)
*p*	Required ATP that is from LET	mol ATP (mol CO_2_)^−1^	3^f^	See main text

CET, cyclic electron transport; LET, linear electron transport.

a The average value for C_4_ species shown by Bellasio & Lundgren (2016), based on the original data of Christin *et al*. (2013).

b Calculated from the information of Griffiths *et al*. (2013) on the average BS : (BS + M) area ratio.

c We use a value for efficiency of PSII electron transport under strictly limiting light conditions to calculate quantum yield (Φ_CO2_) using our model.

d The estimate of light extinction coefficient (*k*) may be uncertain and its actual value depends on the spectrum of light (Bellasio & Griffiths, 2014); however, sensitivity analysis showed that its uncertainty had little impact on the key quantitative estimates of this paper (see Supporting Information Notes S4 and Table S4).

e Leakiness (ϕ) can be very high (close to 1) at low irradiances when ϕ is calculated from the von Caemmerer & Furbank (1999) C_4_ model (see Kromdijk *et al*., 2010; Yin *et al*., 2011). However, that high estimate of ϕ is largely the result of the relatively high flux of day respiration under low‐irradiance conditions. When the effect of day respiration is excluded, the estimated ϕ for low‐irradiance conditions would have a similar value as for normal growth conditions (Yin & Struik, 2012).

f This applies to the NADP‐ME and NAD‐ME subtypes; values of these parameters need to be adjusted accordingly if PEP‐CK decarboxylation is involved (see Methods S1).

The predicted values of β depend strongly on input parameter α, and β is very close to 1 when α is close to zero and *f*_bsPSI_ is high (Fig. 2a). With increasing α, the calculated β declines accordingly, meaning that CET increasingly operates in M cells. However, it appears that there is a limit to the increase of α: for example, α cannot exceed 0.45 if *f*_bsPSI_ is 0.20; otherwise β will become negative (Fig. 2a). This is also illustrated for the calculation of *u* (the fraction of M‐absorbed light that is used for LET) and *v* (the fraction of BS‐absorbed light for LET): when α is beyond 0.45, *v* becomes higher than 1.0 (Fig. 2c).

The calculated fraction of whole‐leaf CET (*f*_CET_) and Φ_CO2_ did not depend on α, nor on *f*_bsPSI_ (Fig. 2d). The modelled *f*_CET_ was 0.537. The modelled Φ_CO2_ was 0.071 mol mol^−1^, comparable with the highest reported Φ_CO2_ for C_4_ species (Ehleringer & Pearcy, 1983). Also, NADPH‐ and ATP‐limited Φ_CO2_ had the same value. These all resulted from the balance between NADPH and ATP production in our model. Our previous analysis (Yin & Struik, 2012) assuming NADPH and ATP co‐limitation resulted in a Φ_2LL_ : Φ_CO2_ ratio comparable with the measured values of this ratio, 11.2–13.1 (Edwards & Baker, 1993), suggesting that the co‐limitation assumption is close to reality.

Our illustration assumed the full operation of the Q cycle, so *H*_CET_ (protons pumped per electron by CET)* *= 2. Kramer & Evans (2011) indicated that *H*_CET_ could be 4 if CET runs in the NAD(P)H dehydrogenase (NDH)‐dependent pathway that generates two more protons per electron (see also Peng *et al*., 2011). We ran this scenario and the obtained Φ_CO2_ was substantially higher than the measured values. The analysis of Kramer & Evans (2011) combined *H*_CET_ of 4 with the proton : ATP ratio of 4.67. Uncertainties exist with regard to this ratio, for C_4_ plants in particular. Furbank *et al*. (1990) assumed the ratio to be 3, while Amthor (2010) indicated 4.67 as being highly unlikely, for C_4_ photosynthesis. Model analysis by matching Φ_CO2_ with quantum yield of PSII photochemistry suggested that a ratio of 4 was most likely (Yin & Struik, 2012), in line with thermodynamic experimental calculations (Steigmiller *et al*., 2008; Petersen *et al*., 2012). It was also shown that the combination of *H*_CET_ of 4 with a proton : ATP ratio of 4.67 resulted in unrealistic estimates of leakiness (Yin & Struik, 2012). These all support our assumption that the proton : ATP ratio is 4. With this ratio, the LET combined with the full operation of the Q cycle generates an ATP : NADPH ratio of 1.5, exactly required by the Calvin cycle (Allen, 2003). This ensures that in the basic form of our model, whole‐leaf LET also satisfies the requirement of NADPH (see below under PEP‐CK subtype and mixed types). However, our model, as explained in Methods S1, works for any combination of these stoichiometric coefficients.

### Prediction of energy budgets in NADP‐ME and NAD‐ME subtypes

Ghannoum *et al*. (2005) reported parameter values of two NADP‐ME species (*Sorghum bicolor*,* Cenchrus ciliaris*) and two NAD‐ME species (*Panicum miliaceum*,* Panicum coloratum*). We used these values as model input to predict a set of output variables (Table 2).

**Table 2:** Model input parameter values for four cases I–IV that represent two NADP‐ME species and two NAD‐ME species as reported by Ghannoum *et al*. (2005), and model predicted output variables for each case, either when no photorespiration nor alternative electron and ATP sinks are assumed (before/) or when photorespiration and alternative electron and ATP sinks are considered (after/) (the equation number, if available in Supporting Information Methods S1, for the corresponding calculation is indicated)

Parameter or variable	NADP‐ME		NAD‐ME	
	*Sorghum bicolor*	*Cenchrus ciliaris*	*Panicum miliaceum*	*Panicum coloratum*
	I	II	III	IV
Input^a^				
Leaf [CHL] (µmol Chl m^−2^)	579	464	424	425
Fraction of [CHL] that is in BS cells (*f*_bsCHL_)	0.33	0.38	0.60	0.59
Fraction of PSI that is in BS cells (*f*_bsPSI_)	0.37	0.39	0.24	0.46
Fraction of PSII that is in BS cells (α)	0.01	0.04	0.17	0.35
Output				
BS : M absorptance ratio (*a*_BS_ : *a*_M_), Eqn C5	0.40/0.40	0.48/0.48	0.92/0.92	0.90/0.90
PSI_CET_ : PSII ratio (*C*_x_ : *T*), Eqn B2	0.81/0.69	0.78/0.66	0.79/0.69	0.94/0.84
Fraction of *C*_*x*_ in BS cells (β), Eqn B10	0.75/0.82	0.78/0.84	0.32/0.33	0.56/0.57
BS : M photosystem ratio	0.31/0.30	0.34/0.33	0.27/0.27	0.73/0.72
BS : M absorptance ratio per photosystem (*k*_BS_ : *k*_M_), Eqn B9	1.30/1.35	1.38/1.43	3.41/3.43	1.23/1.24
Whole‐leaf PSI : PSII ratio, Eqn B12	1.66/1.54	1.62/1.51	1.64/1.54	1.79/1.69
PSI : PSII ratio in BS cells	61.3/56.8	15.8/14.7	2.32/2.18	2.36/2.21
PSI : PSII ratio in M cells	1.05/0.98	1.03/0.96	1.50/1.41	1.48/1.40
Fraction of M‐absorbed light that drives LET (*u*), Eqn A8	0.90/0.94	0.91/0.95	0.74/0.77	0.74/0.77
Fraction of BS‐absorbed light that drives LET (*v*), Eqn A5	0.03/0.03	0.11/0.12	0.56/0.58	0.55/0.58
Whole‐leaf *f*_CET_, Eqn 11	0.54/0.51	0.54/0.51	0.54/0.51	0.54/0.51
*f*_CET_ in BS cells (*f*_CET,BS_), Eqn 11a	0.99/0.99	0.95/0.94	0.63/0.61	0.64/0.62
*f*_CET_ in M cells (*f*_CET,M_), Eqn 11b	0.19/0.13	0.17/0.11	0.43/0.40	0.43/0.39
BS : M total electron flux ratio	0.76/0.79	0.89/0.91	1.08/1.08	1.05/1.06
CET_BS_ : (CET_BS_ + CET_M_)	0.80/0.86	0.83/0.89	0.61/0.62	0.61/0.62
Fraction of NADPH produced in BS cells (*f*_nadph,BS_), Eqn 6	0.01/0.01	0.05/0.06	0.41/0.41	0.40/0.40
Fraction of ATP produced in BS cells (*f*_atp,BS_), Eqn 5	0.35/0.36	0.39/0.39	0.50/0.50	0.49/0.49
Fraction of total BS ATP that is from CET	0.98/0.98	0.92/0.92	0.53/0.51	0.54/0.51
Fraction of ATP that is from LET	0.56/0.59	0.56/0.59	0.56/0.59	0.56/0.59
Φ_CO2_ (mol mol^−1^), Eqn 14	0.071/0.065	0.071/0.065	0.071/0.065	0.071/0.065

BS, bundle sheath; CET, cyclic electron transport; LET, linear electron transport; M, mesophyll.

a The four inputs are from Ghannoum *et al*. (2005); other required inputs are as defined in Table 1.

The predicted BS : M ratio of light absorptance was higher in NAD‐ME species than in NADP‐ME species, in accordance with the difference in the input value of *f*_bsCHL_ (the fraction of chlorophyll in BS cells) between the two subtypes. The predicted *C*_x_ : *T* ratio was *c*. 0.94 in *P. coloratum* and 0.80 in the other three species. The predicted β value was higher in NADP‐ME species (0.75–0.78) than in NAD‐ME species (0.32–0.56). The predicted ratio of total photosystems in BS cells to those in M cells was *c*. 0.3 in NADP‐ME species; but within the two NAD‐ME species, the ratio was much lower in *P. miliaceum* than in *P. coloratum*. In association with this, *k*_BS_/*k*_M_ (the BS : M ratio in the absorptance per unit photosystem) was much higher in *P. miliaceum* than in *P. coloratum*. Although the predicted whole‐leaf PSI : PSII ratio varied little across species (1.62–1.79), in individual cells it varied significantly between the two subtypes: in BS cells, in particular, this ratio was considerably higher in NADP‐ME than in NAD‐ME species. In the two NAD‐ME species, the predicted PSI : PSII ratio in BS cells was somewhat higher than in M cells. In association with this, the predicted *f*_CET_ in BS cells was higher than in M cells. These predictions were surprising given the statement by Takabayashi *et al*. (2005) that the activity of CET and the PSI : PSII ratio should be higher in M cells than in BS cells of NAD‐ME species (see additional analysis in Notes S1 and Table S1). The predicted fraction of M‐absorbed irradiance that is used for LET (*u*) was *c*. 0.90 for NADP‐ME and 0.74 for NAD‐ME species. The predicted fraction of BS‐absorbed irradiance that is used for LET (*v*) was 0.03–0.11 in NADP‐ME species and *c*. 0.55 in the NAD‐ME species. The predicted *v* was largely, but not exclusively, determined by parameter α.

Because of the cell‐type‐specific *u*,* v* and *f*_CET_, energy produced was cell‐type‐specific and differed between subtypes. The fraction of ATP produced in BS cells was 0.35–0.39 in NADP‐ME species and *c*. 0.50 in NAD‐ME species. The fraction of NADPH produced in BS cells was negligible in NADP‐ME species and 0.40 in NAD‐ME species. The ATP produced by CET almost entirely came from BS cells in NADP‐ME species (0.92–0.98, not exactly 1.0 as set by Bellasio & Griffiths, 2014), and the fraction was only *c*. 0.53 in NAD‐ME species.

Regardless of the above differences in the cell‐type‐specific energy budget parameters, predicted *f*_CET_, fraction of ATP that is produced by LET and whole‐leaf Φ_CO2_ were all the same across the species. This confirms the earlier predictions as shown in Fig. 2(d).

### Energy budgets at low ATP : NADPH requirement as occurring in the PEP‐CK subtype

The PEP‐CK subtype, compared with the other subtypes, has a lower ATP : NADPH requirement. Using the model adjusted for this altered requirement (see Eqns 15 and 16 in section 2 of Methods S1), the calculated whole‐leaf *f*_CET_ was very low (0.066) for the case of *n* (ATP produced per oxidation of NADH) = 2.5 and even lower (0.059) for *n *=* *3. As a consequence, the calculated Φ_CO2_ was 0.090 and 0.092 mol mol^−1^, respectively (Table 3), substantially higher than Φ_CO2_ calculated earlier for NADP‐ME and NAD‐ME subtypes. It is equivalent to Φ_CO2_ measured for C_3_ photosynthesis under non‐photorespiratory conditions (Long *et al*., 1993). However, the measured Φ_CO2_ in the PEP‐CK subtype is not higher than that in the NADP‐ME subtype (Ehleringer & Pearcy, 1983). This discrepancy was probably due to factors not yet accounted for in our model, such as photorespiration as a result of possibly high O_2_ released from PSII activity in BS cells (i.e. possibly high α values).

**Table 3:** Calculated quantum yield (Φ_CO2_) and theoretical range of variation in parameter α (fraction of PSII that is in bundle‐sheath (BS) cells) that keeps both *u* (fraction of mesophyll (M) cell‐absorbed light that is used for linear electron transport (LET)) and *v* (fraction of BS cell‐absorbed light that is used for LET) within the range 0–1, in case of the low ATP : NADPH requirement as occurring in the standard PEP‐CK subtype compared with the NADP‐ or NAD‐ME subtypes, when using four cases (I, II, III and IV) of [CHL], *f*_bsCHL_ and *f*_bsPSI_ as shown in Table 2

	Φ_CO2_ (mol mol^−1^)	Range of parameter α			
		I	II	III	IV
Α^a^					
NADP‐, NAD‐ME	0.071^c^	0.000–0.640	0.000–0.670	0.055–0.625	0.115–0.805
PEP‐CK, *n *=* *2.5	0.090	0.325–0.385	0.355–0.400	0.220–0.250	0.430–0.490
PEP‐CK, *n *=* *3.0	0.092	0.325–0.385	0.355–0.400	0.220–0.250	0.430–0.475
Β^b^					
NADP‐, NAD‐ME	0.065^c^	0.000–0.610	0.000–0.655	0.055–0.580	0.145–0.775
PEP‐CK, *n *=* *2.5	0.082	0.325–0.385	0.355–0.400	0.220–0.250	0.430–0.475
PEP‐CK, *n *=* *3.0	0.083	0.340–0.385	0.355–0.400	0.220–0.250	0.445–0.475

*n*, number of ATP produced per oxidation of NADH along the BS mitochondrial electron transport chain.

a When no photorespiration nor alternative electron and ATP sinks are assumed.

b When some photorespiration and alternative electron and ATP sinks (as defined in the text) are considered.

c Also given in Table 2.

To check whether values of α indeed become higher in response to changes in the required ATP : NADPH ratio, we ran the model for a range of α between 0 and 1 combined with each of four sets of three other inputs ([CHL], *f*_bsCHL_ and *f*_bsPSI_) as in Table 2. Note that these inputs may not present the real situation in PEP‐CK species, but are used here to determine the theoretical differences of α as a result of only changes in the required ATP : NADPH ratio in the PEP‐CK subtype relative to the other subtypes. As calculated *u* and *v* change in an opposite direction with a change in α (Fig. 2c), a physiologically relevant range of variation of α was identified as those values of α that keep both *u* and *v* having values between 0 and 1. The identified range of α was much narrower for the PEP‐CK subtype than that for the default NADP‐ or NAD‐ME subtypes (Table 3). As *u* and *v* are factors accounting for the partitioning of light energy between LET and CET, the narrow range of α was simply the result of little requirement for CET in the PEP‐CK subtype. The identified values of α (Table 3) were higher than the corresponding measured α (see Table 2) for the two NADP‐ME and two NAD‐ME species. A higher α means a higher PSII activity in BS and higher LET, which is in line with the observation that leaves in PEP‐CK species have a Chl*a*/*b* ratio lower than that of other C_4_ subtypes and similar to those in C_3_ species, as lower Chl*a*/*b* ratios are linked to lower PSI : PSII activity ratios and in turn with lower CET : LET ratio (Burnell & Hatch, 1988). Our estimate of the negligible *f*_CET_ is also in line with the report of Bräutigam *et al*. (2014) that in contrast to NADP‐ and NAD‐ME species, the transcription of photosynthetic electron transport proteins was unchanged in PEP‐CK species, when compared with C_3_ species where *f*_CET_ is also negligible (Yin *et al*., 2006). The higher projected α value would mean higher photorespiration in BS as a result of higher O_2_ released from PSII in BS cells, which could, at least partly, explain the above mentioned higher‐than‐measured Φ_CO2_ in the PEP‐CK subtype.

### The effect of photorespiration and alternative electron and ATP sinks on energy budgets

Equations 17–20 in section 3 of Methods S1 allow us to quantify the effects of varying amounts of photorespiration and alternative electron and ATP sinks on energy budget and Φ_CO2_. Here for illustration, we assume that the oxygenation to carboxylation ratio is 1 : 20 (Bellasio, 2017), the nitrate‐reduction to carboxylation ratio is 1 : 35 (Kanai & Edwards, 1999) and the day‐respiration to carboxylation ratio is 1 : 40 (data of Yin *et al*., 2011).

The calculated intermediate model parameters changed little for the NADP‐ME and NAD‐ME species, compared to the default calculation (Table 2). Due to photorespiration and nitrate reduction consuming electrons, the calculated Φ_CO2_ decreased slightly to 0.065 mol mol^−1^ (Table 2), virtually identical to the measured Φ_CO2_ for NADP‐ME grass species, but slightly higher than the measured 0.053–0.060 mol mol^−1^ for NAD‐ME species (Ehleringer & Pearcy, 1983). Nitrate reduction hardly consumes ATP; the LET used for supporting nitrate reduction produces ATP that is somewhat more than the ATP consumed by starch synthesis. As a consequence, the required whole‐leaf *f*_CET_ decreased slightly to 0.51 (Table 2).

The inferred physiologically relevant range of α for the PEP‐CK subtype did not change much, compared with the calculation without considering photorespiration and alternative electron and ATP sinks (Table 3). The obtained whole‐leaf *f*_CET_ was 0.060 and 0.053, for *n *=* *2.5 and 3.0, respectively. The corresponding Φ_CO2_ values were 0.082 and 0.083 mol mol^−1^, respectively (Table 3), still much higher than the measured average for the PEP‐CK species, 0.064 mol mol^−1^ (Ehleringer & Pearcy, 1983).

Our calculation was based on the consideration that the function of the PEP‐CK cycle depends exclusively on the provision of ATP by a parallel function of mitochondrial electron transport (Hatch, 1987; Burnell & Hatch, 1988). Although the parallel mitochondrial respiration to fuel PEP‐CK is supported by the observation that O_2_ uptake in the light was much higher in PEP‐CK than in other C_4_ species (Furbank & Badger, 1982), it might represent the maximally efficient case. Uncertainties exist on the extent of OAA that is converted to malate in M cells in the PEP‐CK subtype (Ishikawa *et al*., 2016). Koteyeva *et al*. (2015) reviewed several studies, in which a variable and low activity of NAD‐ME was observed among PEP‐CK species, and discussed several alternatives for generating ATP in BS cells. For example, ATP supply possibly occurs through the oxidation of triose phosphate (triose‐P) to 3‐PGA in the BS cytoplasm (Kanai & Edwards, 1999), which would increase the chloroplastic energy cost. In such a case, the theoretical Φ_CO2_ would be lower than the one we obtained so far. The gaps between measured and theoretical Φ_CO2_ in the PEP‐CK subtype as well as in the NAD‐ME subtype could also be due to somewhat higher photorespiration for these subtypes, as a result of either higher α or possibly higher leakiness or both. Possibly, higher leakiness in these subtypes is related to the location of decarboxylation, mitochondria for the NAD‐ME and in cytosol for the PEP‐CK subtype, where decarboxylated CO_2_ could easily escape from fixation by Rubisco, compared with the CO_2_ decarboxylated in chloroplasts for NADP‐ME species. The relative importance of the three reasons (using chloroplast ATP to fuel PEP‐CK, high O_2_, and high leakiness) in explaining the discrepancy between theoretical and measured Φ_CO2_ for the PEP‐CK subtype may be species‐specific, and its full elucidation is beyond the scope of our analysis but warrants further experimental studies.

### Strategies to satisfy the cell‐type specific energy supply:demand balance

Next we examined if cell‐type‐specific production and requirement of NADPH and ATP are in balance. Cell‐type‐specific requirement of NADPH and ATP, as given in Fig. S1, is based on the simplest scenario that (1) 50% of the reduction of 3‐PGA occurs in M cells and the remaining 50% in BS cells, and (2) there is no leakiness, nor photorespiration or alternative energy‐using sink. When considering leakiness and allowing for a variable proportion of the 3‐PGA reduction occurring in the BS cells (γ), we formulated algorithms for calculating cell‐type‐specific NADPH and ATP requirement per CO_2_ assimilated for the three subtypes (Table 4). By setting the BS : total ratio in NADPH or ATP production (Table 2) equal to the BS : total ratio in NADPH or ATP requirement, one can solve for γ given that a leakiness (ϕ) of 0.16 (Yin & Struik, 2012) has been used for calculating the output variables in Table 2. The solved γ (which ensures that cell‐type‐specific NADPH and ATP production meets cell‐type‐specific NADPH and ATP requirements) in any of the four species was not equal to the previously assumed value 0.5 (Table 5). More surprisingly, the solved γ for NADPH was not equal to but higher in the NADP‐ME species and lower in the NAD‐ME species than that solved for ATP (Table 5). Photorespiration and other electron and ATP sinks altered the results only little (Table 5). The reduction of 3‐PGA involves an intercellular shuttle (Hatch, 1987): 3‐PGA may move from BS cells to M cells, and is first transformed to 1,3‐bisphosphoglycerate by phosphoglycerate kinase (the step which requires ATP) and then reduced to triose‐P by glyceraldehyde 3‐phosphate dehydrogenase (the step that requires NADPH), and triose‐P may move back to BS cells. Higher γ for ATP than for NADPH, as calculated for NAD‐ME species, indicates the possibility that relative to the second step, the first step of 3‐PGA reduction occurs more in BS than in M cells, which suggests an interesting question for experimental research such as carried out by Arrivault *et al*. (2017) to confirm. However, it is hard to reconcile with a lower γ for ATP than for NADPH, as calculated for NADP‐ME species.

**Table 4:** Theoretical cell‐type‐specific NADPH and ATP requirements per CO_2_ assimilated in three classical (i.e. NADP‐ME, NAD‐ME and PEP‐CK) C_4_ subtypes (A), and some supplementary or mixing types (B)

	M	BS	BS : total ratio
A			
NADP‐ME			
NADPH	(1 + ϕ) + 2(1 − γ) + *x*_1_	−(1 + ϕ) + 2γ + *x*_2_	(2γ − ϕ−1 + *x*_2_) : (2 + *x*_1_ + *x*_2_)
ATP	2(1 + ϕ) + 2(1 − γ) + *x*_3_	1 + 2γ + *x*_4_	(2γ + 1 + *x*_4_) : [3 + 2(1 + ϕ) + *x*_3_ + *x*_4_]
NAD‐ME			
NADPH	2(1 − γ) + *x*_1_	2γ + *x*_2_	(2γ + *x*_2_) : (2 + *x*_1_ + *x*_2_)
ATP	2(1 + ϕ) + 2(1 − γ) + *x*_3_	1 + 2γ + *x*_4_	(2γ+1 + *x*_4_) : [3 + 2(1 + ϕ) + *x*_3_ + *x*_4_]
PEP‐CK			
NADPH	*a*(1 + ϕ) + 2(1 − γ) + *x*_1_	2γ + *x*_2_	(2γ + *x*_2_) : [2 + *a*(1 + ϕ) + *x*_1_ + *x*_2_]
ATP	2*a*(1 + ϕ) + 2(1 − γ) + *x*_3_	1 + 2γ + *x*_4_	(2γ + 1 + *x*_4_) : [3 + 2*a*(1 + ϕ) + *x*_3_ + *x*_4_]
B			
‘Aspartate–malate’			
NADPH	2(1 − γ) + *x*_1_	2γ + *x*_2_	(2γ + *x*_2_) : (2 + *x*_1_ + *x*_2_)
ATP	2(1 + ϕ) + 2(1 − γ) + *x*_3_	1 + 2γ + *x*_4_	(2γ + 1 + *x*_4_) : [3 + 2(1 + ϕ) + *x*_3_ + *x*_4_]
NADP‐ME + ‘Aspartate–malate’			
NADPH	η(1 + ϕ) + 2(1 − γ) + *x*_1_	−η(1 + ϕ) + 2γ + *x*_2_	[2γ − η(1 + ϕ) + *x*_2_] : (2 + *x*_1_ + *x*_2_)
ATP	2(1 + ϕ) + 2(1 − γ) + *x*_3_	1 + 2γ + *x*_4_	(2γ + 1 + *x*_4_) : [3 + 2(1 + ϕ) + *x*_3_ + *x*_4_]
‘Pure’ PEP‐CK			
NADPH	2(1 − γ) + *x*_1_	2γ + *x*_2_	(2γ + *x*_2_) : (2 + *x*_1_ + *x*_2_)
ATP	2(1 − γ) + *x*_3_	1 + 2γ + (1 + ϕ) + *x*_4_	(2γ + 1 + ϕ + *x*_4_) : [4 + ϕ + *x*_3_ + *x*_4_]
NADP‐ME + PEP‐CK			
NADPH	η(1 + ϕ) + 2(1 − γ) + *x*_1_	−η(1 + ϕ) + 2γ + *x*_2_	[2γ − η(1 + ϕ) + *x*_2_] : (2 + *x*_1_ + *x*_2_)
ATP	2 η(1 + ϕ) + 2(1 − γ) + *x*_3_	(1 − η)(1 + ϕ) + 1 + 2γ + *x*_4_	[2γ + 1 + (1 − η)(1 + ϕ) + *x*_4_] : [3 + (1 + η)(1 + ϕ) + *x*_3_ + *x*_4_]
NAD‐ME + PEP‐CK			
NADPH	2(1 − γ) + *x*_1_	2γ + *x*_2_	(2γ + *x*_2_) : (2 + *x*_1_ + *x*_2_)
ATP	2η(1 + ϕ)+2(1 − γ) + *x*_3_	(1 − η)(1 + ϕ) + 1 + 2γ + *x*_4_	[2γ+1 + (1 − η)(1 + ϕ) + *x*_4_] : [3 + (1 + η)(1 + ϕ) + *x*_3_ + *x*_4_]
NADP‐ME + ‘Aspartate–malate’ + PEP‐CK			
NADPH	η_1_(1 + ϕ) + 2(1 − γ) + *x*_1_	−η_1_(1 + ϕ) + 2γ + *x*_2_	[2γ − η_1_(1 + ϕ) + *x*_2_] : (2 + *x*_1_ + *x*_2_)
ATP	2(η_1_ + η_2_)(1 + ϕ) + 2(1 − γ) + *x*_3_	(1 − η_1_ − η_2_)(1 + ϕ) + 1 + 2γ + *x*_4_	[2γ+1 + (1 − η_1_ − η_2_)(1 + ϕ) + *x*_4_] : [3 + (1 + η_1_ + η_2_)(1 + ϕ) + *x*_3_ + *x*_4_]

Note that (i) values in the formulae in A here for the BS : total ratio will become those in the table shown in Supporting Information Fig. S1 for the simplest scenario where ϕ = 0, γ = 0.5, *a *=* *0.25 and *x*_1_, *x*_2_, *x*_3_ and *x*_4_ = 0; and (ii) NADPH or ATP requirements in mesophyll (M) and bundle‐sheath (BS) cells of the mixed types in B here are formulated as the weighted average of the requirements in the involved decarboxylating routes.

ϕ, leakiness.

γ, fraction of NADPH or ATP that is consumed in BS cells for 3‐phosphoglycerate (3‐PGA) reduction.

*a*, fraction of oxaloacetate (OAA) that is reduced in M chloroplasts to malate; 1 − *a* is then the remaining fraction of OAA that is directly decarboxylated in the BS cytosol (applicable to the PEP‐CK subtype; see also Fig. S1c). Assuming that ATP required for direct decarboxylation of OAA in the BS cytosol exclusively comes from NADH oxidation in the respiratory electron transport chain, which is coupled with malate decarboxylation in BS mitochondria, *a* can be solved from: *na* = 1(1 − *a*), so, *a *=* *1/(1 + *n*), where *n* refers to mol ATP produced per oxidation of NADH in the mitochondrial electron transport chain and 1 refers to 1 mol ATP required for decarboxylation of 1 mol OAA by PEP‐CK (Kanai & Edwards, 1999). As *n* is either 2.5 (Hinkle *et al.,* 1991) or 3.0 (Ferguson, 1986), *a* can be solved as either 0.286 or 0.250.

*x*_1_, *x*_2_, *x*_3_ and *x*_4_ in the table are required when photorespiration and alternative electron and ATP sinks (starch synthesis and nitrate reduction) are considered in the analysis. We use ν_o/c_, ν_n/c_ and ν_r/c_ to refer to the ratios of oxygenation, nitrate reduction and day respiration to carboxylation, respectively. It is assumed in the analysis that (i) in the photorespiratory carbon oxidation (PCO) cycle, only NADPH and ATP consumption during the 3‐PGA reduction phase (i.e. 1.5ν_o/c_ NADPH and 1.5ν_o/c_ ATP, von Caemmerer 2000) is partitioned between BS and M cells, whereas the remaining 0.5ν_o/c_ NADPH and 2ν_o/c_ ATP consumption by the PCO cycle takes place in BS cells; and (ii) nitrate reduction predominantly takes place in the M cells whereas starch synthesis predominantly takes place in the BS cells (Furbank *et al*., 1990; Kanai & Edwards, 1999; Majeran *et al*., 2005; Majeran & van Wijk, 2009; Friso *et al*., 2010). Based on these, *x*_1_ = 1.5ν_o/c_(1 − γ) + 5ν_n/c_, *x*_2_ = 1.5ν_o/c_γ + 0.5ν_o/c_, *x*_3_ = 1.5ν_o/c_(1 − γ) + ν_n/c_ and *x*_4_ = 1.5ν_o/c_γ + 2ν_o/c_ + 0.167(1−0.5ν_o/c_ − ν_r/c_).

η, fraction of OAA that follows the primary decarboxylation pathway (applied only to the double mixed decarboxylation types).

η_1_, fraction of C_4_ acids following the primary NADP‐ME route; η_2_, the fraction following the ‘aspartate–malate’ route (applied only to a triple mixed decarboxylation type).

**Table 5:** Calculated energy budget expressed as the ratio of the amount in bundle‐sheath (BS) cells to the total in the two cell types, for two NADP‐ME species (I, II) and two NAD‐ME species (III, IV) as reported by Ghannoum *et al*. (2005), either when no photorespiration nor alternative electron and ATP sinks are assumed (before/) or when photorespiration and alternative electron and ATP sinks are considered (after/)

	I	II	III	IV
Fraction of energy production in BS cells^a^				
NADPH	0.01/0.01	0.05/0.06	0.41/0.41	0.40/0.40
ATP	0.35/0.36	0.39/0.39	0.50/0.50	0.49/0.49
Calculated required fraction γ^b^				
NADPH	0.59/0.56	0.63/0.61	**0.41/0.43**	**0.40/0.42**
ATP	**0.43/0.38**	**0.54/0.46**	0.83/0.76	0.80/0.74
Required η in the ‘aspartate–malate’ mechanism^c^	**0.73/0.68**	**0.84/0.73**	d	d
Required η in the ‘PEP‐CK’ mechanism^c^	1.70/1.88^e^	1.39/1.56^e^	**0.78/0.75**	**0.80/0.78**
Required values in the triple decarboxylation pathway^f^				
η_1_	0.67/0.75	0.57/0.65		
η_2_	0.27/0.30	0.23/0.26		

The most likely estimates for the fraction of 3‐PGA reduction in BS cells and for the fraction of a mixed decarboxylation (see Discussion) are shown in bold type.

a This fraction, as an output (see Table 2) of our analytical model, depends on C_4_ subtypes, among which cell‐type‐specific ATP requirement differs. So, in principle, this fraction calculated by the model varies mathematically once a mixed decarboxylation pathway is involved. However, the modelled results for this fraction only varied after two decimal digits, and this minor change is not given in this Table.

b γ, required fraction of NADPH or ATP consumption during the reductive phase of the Calvin cycle that takes place in the BS cells, if a secondary decarboxylation pathway is not engaged.

c η, required fraction of OAA that follows the primary decarboxylation pathway if a secondary decarboxylation pathway (either the ‘aspartate–malate’ or the ‘PEP‐CK’ pathway) is also engaged.

d No η values were calculated here in Cases III and IV (i.e. two NAD‐ME species) because the cell‐type‐specific NADPH and ATP requirements for the supplementary ‘aspartate–malate’ mechanism are the same as those for the NAD‐ME species (Table 4).

e The calculated η values for Cases I and II here are above 1.0, which is physiologically impossible; they are presented here merely to show the results of mathematical calculation (see Discussion).

f The calculated values of η_1_ and η_2_ (η_1_ = fraction of C_4_ acids following the primary NADP‐ME route; η_2_, the fraction following the ‘aspartate–malate’ route) in a triple decarboxylation pathway in Cases I and II of two NADP‐ME species (see Discussion).

It has long been observed that in NADP‐ME species, some OAA is converted into aspartate in M cells (Hatch, 1971; Gutierrez *et al*., 1974; Chapman & Hatch, 1981; Shieh *et al*., 1982; Meister *et al*., 1996), as occurs in NAD‐ME species. This would affect the cellular energy balance in NADP‐ME species. If aspartate is translocated to BS cells, it must be transaminated back to OAA, reduced to malate and then decarboxylated. In *Flaveria bidentis*, the reduction of OAA takes place in BS chloroplasts, although mitochondrial NAD‐MDH could also play a role (Meister *et al*., 1996; Furbank, 2011). Regardless of whether the OAA reduction occurs in BS chloroplasts or mitochondria, the NADPH requirement in M and BS cells by this specific ‘aspartate‐malate’ mechanism differs from that in the classical NADP‐ME subtype, but is the same as in the NAD‐ME subtype (Table 4). Let η be the fraction of OAA following the primary NADP‐ME route and the remaining (1‐ η) be the fraction following the secondary ‘aspartate–malate’ mechanism; the NADPH requirements in M and BS cells and the BS : total ratio in NADPH requirement for the mixed NADP‐ME and ‘aspartate–malate’ pathway could thus be formulated, depending on η (Table 4). As cellular ATP requirements are the same for standard NADP‐ME and NAD‐ME subtypes (Table 4), the BS : total ratio for ATP requirement remains unaltered. We now can solve for η if cell‐type‐specific NADPH and ATP production meet cell‐type‐specific NADPH and ATP requirements in the two NADP‐ME species. If γ for NADPH is the same as for ATP as earlier estimated (i.e. 0.38–0.54; Table 5), the solved value of η is 0.68 or 0.73 for *Sorghum bicolor*, and 0.73 or 0.84 for *Cenchrus ciliaris*, depending on whether photorespiration and alternative electron and ATP sinks were considered (Table 5). This means that 16–32% of OAA was predicted to follow the ‘aspartate–malate’ pathway, comparable with the early observation for maize that *c*. 25% of the initial carbon label is partitioned to aspartate (Hatch, 1971). However, the values of η also depended on structural parameters (Notes S2; Table S2), which differ among C_4_ species (Hatterslay, 1984). A higher value, up to 50%, for the ‘aspartate–malate’ pathway was observed for *Flaveria bidentis* (Meister *et al*., 1996).

The above analysis suggests that C_4_ plants can engage: (i) 3‐PGA/triose‐P shuttle between M and BS cells, and (ii) mixed C_4_–acid decarboxylation pathways, to achieve cell‐type‐specific demand : supply balances in terms of both ATP and NADPH. In addition, leakiness, photorespiration, and alternative electron and ATP sinks also play a minor role.

### Further analysis of effects of mixed decarboxylation on cellular energy balance

The ‘aspartate–malate’ pathway is not the only form of a mixed decarboxylation pathway in NADP‐ME species. The OAA in BS cells transaminated from aspartate may not be reduced to malate by NAD(P)‐MDH, but could be directly decarboxylated in BS cytosol by PEP‐CK. It has been observed in maize that PEP‐CK is active in supporting the aspartate‐dependent decarboxylation (e.g. Majeran *et al*., 2005; Sommer *et al*., 2012; Koteyeva *et al*., 2015). ATP required for this direct decarboxylation could come from BS chloroplasts. To analyse whether PEP‐CK can be used as an alternative mixed decarboxylating mechanism, it is necessary to first model the energy production of the ‘pure’ PEP‐CK type that acts alone without using mitochondrial electron transport to provide ATP (see section 2 of Methods S1).

We run the model with four cases (i.e. I–IV) as defined in Table 2, again merely to check the theoretical consequences of only changes in the required NADPH : ATP ratio for the ‘pure’ PEP‐CK type relative to the other subtypes. We consider photorespiration and nitrate reduction and starch synthesis as defined earlier. Using the same criteria as stated before resulted in a wider physiologically relevant range of α (Table 6), compared with its range for the standard PEP‐CK subtype (Table 3). Other parameters associated with energy production were also generated by the model (Table 6). The modelled whole‐leaf *f*_CET_ was 0.34 and Φ_CO2_ was 0.078 mol mol^−1^, both somewhere between the values for the standard PEP‐CK subtype and the malic enzyme subtypes. This was because the minimum of 1 mol ATP required for CCM in the ‘pure’ PEP‐CK type is between that for the standard PEP‐CK subtype (0.5 or 0.572 mol ATP) and that for the malic enzyme subtypes (2 mol ATP).

**Table 6:** Calculated theoretical range of variation in parameter α (fraction of PSII that is in bundle‐sheath (BS) cells) that keeps both *u* (fraction of M cell‐absorbed light that is used for linear electron transport (LET)) and *v* (fraction of BS cell‐absorbed light that is used for LET) within the range 0–1, and the corresponding range of other parameters when ATP requirement is as for the ‘pure’ PEP‐CK type, using four cases (I, II, III and IV) of [CHL], *f*_bsCHL_ and *f*_bsPSI_ as shown in Table 2

	I	II	III	IV
Output				
Relevant range of α	0.10–0.51	0.15–0.54	0.12–0.42	0.29–0.66
PSI_CET_ : PSII ratio (*C*_*x*_ : *T*)	0.37–0.34	0.37–0.34	0.14–0.63	0.33–0.35
Fraction of *C*_*x*_ that is in BS cells (β)	0.99–0.03	0.95–0.03	0.98–0.03	0.96–0.04
BS : M photosystem ratio	0.33–0.76	0.39–0.84	0.22–0.45	0.59–1.25
*k*_BS_ : *k*_M_	1.21–0.52	1.23–0.57	4.29–2.05	1.52–0.72
*f*_CET_ in BS cells (*f*_CET,BS_)	0.81–0.02	0.74–0.02	0.59–0.05	0.59–0.03
*f*_CET_ in M cells (*f*_CET,BS_)	0.01–0.44	0.03–0.46	0.00–0.56	0.00–0.57
Fraction of NADPH produced in BS cells (*f*_nadph,BS_)	0.12–0.35	0.17–0.40	0.36–0.59	0.35–0.59
Fraction of ATP produced in BS cells (*f*_atp,BS_)	0.34–0.26	0.37–0.30	0.52–0.44	0.51–0.43
Whole‐leaf *f*_CET_	0.34	0.34	0.34	0.34
Φ_CO2_ (mol mol^−1^)	0.078	0.078	0.078	0.078
γ^a^				
for NADPH	0.12–0.37	0.17–0.42	0.37–0.63	0.37–0.63
for ATP	−0.31 to −0.46	−0.24 to −0.39	0.05 to −0.10	0.04 to −0.13

a γ, required fraction of NADPH or ATP consumption during the reductive phase of the Calvin cycle that takes place in the BS cells.

Cell‐type‐specific energy requirements for the ‘pure’ PEP‐CK type can easily be defined (Table 4). The BS : total ATP requirement ratio for the simplest scenario is 3 : 4 for this type, much higher than that for any standard C_4_ subtype (Fig. S1). As done earlier, one can solve for γ by setting the BS : total production ratio equal to the BS : total requirement ratio. The value of γ solved for NADPH was reasonable whereas that for ATP was mostly negative (Table 6). Only for Cases III and IV where *f*_bsCHL_ (the fraction of Chl in BS cells) was relatively high, was the calculated γ for ATP positive; however, these γ values for ATP were lower than those for NADPH, which is again hard to reconcile with the fact that the first step of 3‐PGA reduction requires ATP and its second step requires NADPH. Adjusting *f*_bsCHL_ to 0.7 (as observed for PEP‐CK species, Ku *et al*., 1974) or to be extremely high (0.9), or decreasing areas of M cells in Fig. 1, increased the fraction of ATP and NADPH produced in BS cells but a lower γ for ATP than for NADPH was always obtained. The negative γ or a lower γ for ATP than for NADPH is impossible physiologically but its mathematical occurrence simply indicates that the high ATP requirement in the BS cells of the ‘pure’ PEP‐CK type is impossible to achieve. Using numerical simulation, Wang *et al*. (2014) also suggested that the ‘pure’ PEP‐CK type is almost impossible to realise in terms of the energy requirement in BS cells. In the ‘pure’ PEP‐CK type, PEP returns directly to M cells without pyruvate regeneration by reaction with alanine aminotransferase, and there will be an imbalance in NH_2_ flux between M and BS compartments (Furbank, 2011). The impossibility of satisfying the ATP : NADPH requirement and the imbalance in NH_2_ flux between M and BS compartments explain why such a PEP‐CK‐only mechanism does not appear in nature, as noted by von Caemmerer & Furbank (2016).

Next, we examine to what extent PEP‐CK could be used in NADP‐ME and NAD‐ME subtypes to form a mixed decarboxylation mechanism. We will call these ‘NADP‐ME + PEP‐CK’ and ‘NAD‐ME + PEP‐CK’ types, respectively. Again let η be the fraction of OAA following the primary NADP‐ME (or NAD‐ME) route to formulate the NADPH or ATP requirements in each cell type (Table 4). How our model accommodates energy production in these mixed types is described in section 4 of Methods S1.

For the case of the mixed ‘NADP‐ME + PEP‐CK’ type, the fractions of both NADPH and ATP required in BS cells depend on η (Table 4). One can expect to solve simultaneously for γ and η by setting the BS : total requirement ratio equal to the BS : total production ratio for NADPH as well as for ATP. The value of solved η was 1.70 or 1.88 for *S. bicolor* (Case I) and 1.39 or 1.56 for *C. ciliaris* (Case II) (Table 5; see Notes S3 and Table S3 for additional analysis). A fraction above 1.0 is physiologically impossible, and its occurrence was the mathematical reflection that PEP‐CK cannot be a supplementary decarboxylation pathway in these species. Indeed, it has been reported that sorghum does not have PEP‐CK but a high level of aspartate transaminase and alanine transaminase (Gutierrez *et al*., 1974; Voznesenskaya *et al*., 2006; Koteyeva *et al*., 2015), supporting our earlier analysis that the physiologically sensible value of η was obtained if the ‘aspartate–malate’ mechanism is the secondary pathway (Table 5).

For the mixed ‘NAD‐ME + PEP‐CK’ type, the fraction of only ATP (not NADPH) required in BS cells depends on η (Table 4). Earlier we have shown that for the two NAD‐ME species the calculated fraction γ for NADPH was lower than the solved value of γ for ATP if there was no secondary decarboxylation (Table 5). Although this can be reconciled by the possibility that relative to the second step of 3‐PGA reduction (which requires NADPH), its first step (which requires ATP) occurs more in BS than in M cells, the probablility of a different γ for NADPH and ATP is low. If PEP‐CK is engaged as the secondary pathway, γ for ATP can be made equal to that for NADPH (*c*. 0.40–0.43, Table 5), and one can solve for η by setting the BS : total requirement ratio equal to the BS : total production ratio for ATP. This gave a value of η of 0.75–0.80 (Table 5). However, again the solved value of η depended on the structural parameters (Notes S3; Table S3). PEP‐CK activity is generally low in NAD‐ME species, but substantial amounts of PEP‐CK have been reported in *Eragrostis nutans* (Koteyeva *et al*., 2015) and in old leaves of *Cleome gynandra* (Sommer *et al*., 2012). Our model calculated that involvement of PEP‐CK has some consequences for Φ_CO2_. If η was changed from 1 to 0.75, the required whole‐leaf *f*_CET_ decreased from 0.510 (Table 2) to 0.475, and Φ_CO2_ increased from 0.065 (Table 2) to 0.068 mol mol^−1^.

The above analysis indicates that NADP‐ME and NAD‐ME subtypes, if involving a mixed pathway, would need to have ‘aspartate–malate’ and PEP‐CK, respectively, as their secondary decarboxylation route. However, maize, a consummate NADP‐ME plant, has appreciable amounts of PEP‐CK (e.g. Sommer *et al*., 2012; Koteyeva *et al*., 2015). This suggests a possible triple decarboxylation pathway (also see Wang *et al*., 2014). Let η_1_ be the fraction of C_4_ acids following the primary NADP‐ME route, η_2_ be the fraction for the ‘aspartate–malate’ route and the remaining (1 − η_1_ − η_2_) be the fraction for the ‘PEP‐CK’ route, to formulate the NADPH or ATP requirements in M and BS cells of the triple decarboxylation pathway (Table 4). Three unknowns (η_1_, η_2_, γ) cannot be solved from the aforementioned two equations arising from setting the BS : total requirement ratio equal to the BS : total production ratio for NADPH and ATP, respectively. Here, we rely on the additional information on the BS : M ratio of NADP‐MDH, which was reported to be up to 0.39 in maize (Majeran *et al*., 2005), which can be related to the η_2_ : η_1_ ratio. The value of η_1_ we obtained was 0.57–0.75, and η_2_ was 0.23–0.30 (Table 5), which are more realistic than those calculated earlier assuming the double ‘NADP‐ME + PEP‐CK’ decarboxylation for these species. The corresponding γ was 0.39–0.42 (result not shown in Table 5). Clearly, parameters for the triple decarboxylation pathway are more difficult to solve, and the results here are uncertain and presented merely as an indication of this route.

### Concluding remarks

The input parameters of our model are amenable to direct experimental measurement. The modelled whole‐leaf *f*_CET_ was *c*. 0.50 for NADP‐ME and NAD‐ME subtypes and only *c*. 0.05 for the standard PEP‐CK subtype. The CET accounted for almost 100% of the electron flux in BS cells and only *c*. 10% in M cells for the NADP‐ME subtype having negligible PSII in BS cells, but *c*. 60% in BS cells and *c*. 40% in M cells for the NAD‐ME subtype. Associated with its negligible CET, the standard PEP‐CK subtype was modelled to have the highest proportion of PSII in BS cells.

The calculated fractions of NADPH and ATP production in the BS cells were used to match the fractions required for the operation of the Calvin cycle and the CCM cycle. The analysis suggested that the ‘pure’ PEP‐CK type did not seem to exist because ATP or ATP : NADPH requirements in BS cells were impossible to fulfil. However, there were uncertainties in structural parameters, and sensitivity analyses with respect to these parameters suggested that the NAD‐ME subtype had a higher phenotypic plasticity than the NADP‐ME subtype (see Notes S1–S3). It was shown that some C_4_ acids followed a secondary decarboxylation route, which was obligatory (in the ‘aspartate–malate’ route) in the NADP‐ME subtype, but facultative (in the PEP‐CK route, if any) in the NAD‐ME subtype. With such mixed decarboxylation, the fraction of 3‐PGA reduction in BS cells was not 0.5 as previously assumed, but *c*. 0.4, comparable with the result of Majeran *et al*. (2005) that 3‐PGA reduction occurs more in the M cells. However, this fraction may vary with species.

Recent literature suggests the necessity of mixed decarboxylation mechanisms, probably under the fluctuating environmental conditions for the NADPH : ATP ratio to be flexible in each cell type (e.g. Furbank, 2011; Bellasio & Griffiths, 2014; Stitt & Zhu, 2014). We demonstrate that a mixed decarboxylation is needed to achieve a balanced NADPH and ATP budget in both M and BS cells under steady‐state light conditions. The modelled Φ_CO2_ assuming that energy supply matches demand was close to the measured values, at least for the NADP‐ME subtype, and Φ_CO2_ was shown to be theoretically higher in types involving PEP‐CK and was highest in the standard PEK‐CK subtype. Therefore, mixed types involving PEP‐CK may alleviate low Φ_CO2_ and improve canopy productivity of NADP‐ME crop plants. However, the trade‐off between low energy costs and possibly high photorespiratory losses in the PEP‐CK subtype needs more quantitative analyses.

## Author contributions

X.Y. conceived the research, developed the model and conducted the analyses; X.Y. and P.C.S. wrote the article.

## Supporting information

Please note: Wiley Blackwell are not responsible for the content or functionality of any Supporting Information supplied by the authors. Any queries (other than missing material) should be directed to the *New Phytologist* Central Office.

**Fig. S1** Decarboxylation mechanism and minimum cell‐type‐specific energy requirements of three standard subtypes of C_4_ photosynthesis.
**Table S1** Effect of structure parameters on modelled fractions of CET in NAD‐ME species
**Table S2** Effect of structure parameters on the modelled requirement of the ‘aspartate–malate’ mechanism as the secondary decarboxylating pathway in NADP‐ME species
**Table S3** Effect of structure parameters on the modelled requirement of the ‘PEP‐CK’ mechanism as the secondary decarboxylating pathway
**Table S4** Effect of light extinction coefficient *k* on the modelled requirement of the secondary decarboxylating pathway
**Methods S1** Analytical model for cell‐type‐specific electron transport.
**Methods S2** FST codes of our model for NADPH and ATP production and quantum yield.
**Notes S1** Effect of structural parameters on modelled fractions of CET in NAD‐ME species.
**Notes S2** Effect of structural parameters on the estimated requirement of the ‘aspartate–malate’ mechanism as the secondary decarboxylating pathway in NADP‐ME species.
**Notes S3** Effect of structural parameters on the estimated requirement of the ‘PEP‐CK’ mechanism as the secondary decarboxylating pathway in NADP‐ME and NAD‐ME species.
**Notes S4** Effect of light extinction coefficient k on the estimated requirement of the secondary decarboxylating pathway in NADP‐ME and NAD‐ME species.
