# Supplementary material for: The energy budget in C4 photosynthesis: insights from a cell‐type‐specific electron transport model
Source: New Phytol. 2018 Mar 9;218(3):986–98. doi: 10.1111/nph.15051 (PMC5947737; doi:10.1111/nph.15051)
Supplement: Supplementary file 1 — Fig. S1 Decarboxylation mechanism and minimum cell‐type‐specific energy requirements of three standard subtypes of C4 photosynthesis. Table S1 Effect of structure parameters on modelled fractions of CET in NAD‐ME species Table S2 Effect of structure parameters on the modelled requirement of the ‘aspartate–malate’ mechanism as the secondary decarboxylating pathway in NADP‐ME species Table S3 Effect of structure parameters on the modelled requirement of the ‘PEP‐CK’ mechanism as the secondary decarboxylating pathway Table S4 Effect of light extinction coefficient k on the modelled requirement of the secondary decarboxylating pathway Methods S1 Analytical model for cell‐type‐specific electron transport. Methods S2 FST codes of our model for NADPH and ATP production and quantum yield. Notes S1 Effect of structural parameters on modelled fractions of CET in NAD‐ME species. Notes S2 Effect of structural parameters on the estimated requirement of the ‘aspartate–malate’ mechanism as the secondary decarboxylating pathway in NADP‐ME species. Notes S3 Effect of structural parameters on the estimated requirement of the ‘PEP‐CK’ mechanism as the secondary decarboxylating pathway in NADP‐ME and NAD‐ME species. Notes S4 Effect of light extinction coefficient k on the estimated requirement of the secondary decarboxylating pathway in NADP‐ME and NAD‐ME species. [file NPH-218-986-s001.pdf]

New Phytologist

***New Phytologist* Supporting Information**

Article title: The energy budget in C_4 photosynthesis: insights from a cell-type specific electron transport model

Authors: Xinyou Yin & Paul C. Struik

Article acceptance date: 16 January 2018

The following Supporting Information is available for this article:

Fig. S1 The decarboxylation mechanism and minimum cell-type specific energy requirements of three standard subtypes of C_4 photosynthesis

Methods S1 The analytical model for cell-type specific electron transport

Methods S2 FST codes of our model for NADPH and ATP production and quantum yield

Notes S1 The effect of structure parameters on modelled fractions of CET in NAD-ME species

Notes S2 The effect of structure parameters on the estimated requirement of the "aspartate-malate" mechanism as the secondary decarboxylating pathway in NADP-ME species

Notes S3 The effect of structure parameters on the estimated requirement of the "PEP-CK" mechanism as the secondary decarboxylating pathway in NADP-ME and NAD-ME species

Notes S4 The effect of light extinction coefficient *k* on the estimated requirement of the secondary decarboxylating pathway in NADP-ME and NAD-ME species

Table S1 The effect of structure parameters on modelled fractions of CET in NAD-ME species

Table S2 The effect of structure parameters on the modelled requirement of the "aspartate-malate" mechanism as the secondary decarboxylating pathway in NADP-ME species

Table S3 The effect of structure parameters on the modelled requirement of the "PEP-CK" mechanism as the secondary decarboxylating pathway

Table S4 The effect of light extinction coefficient *k* on the modelled requirement of the secondary decarboxylating pathway

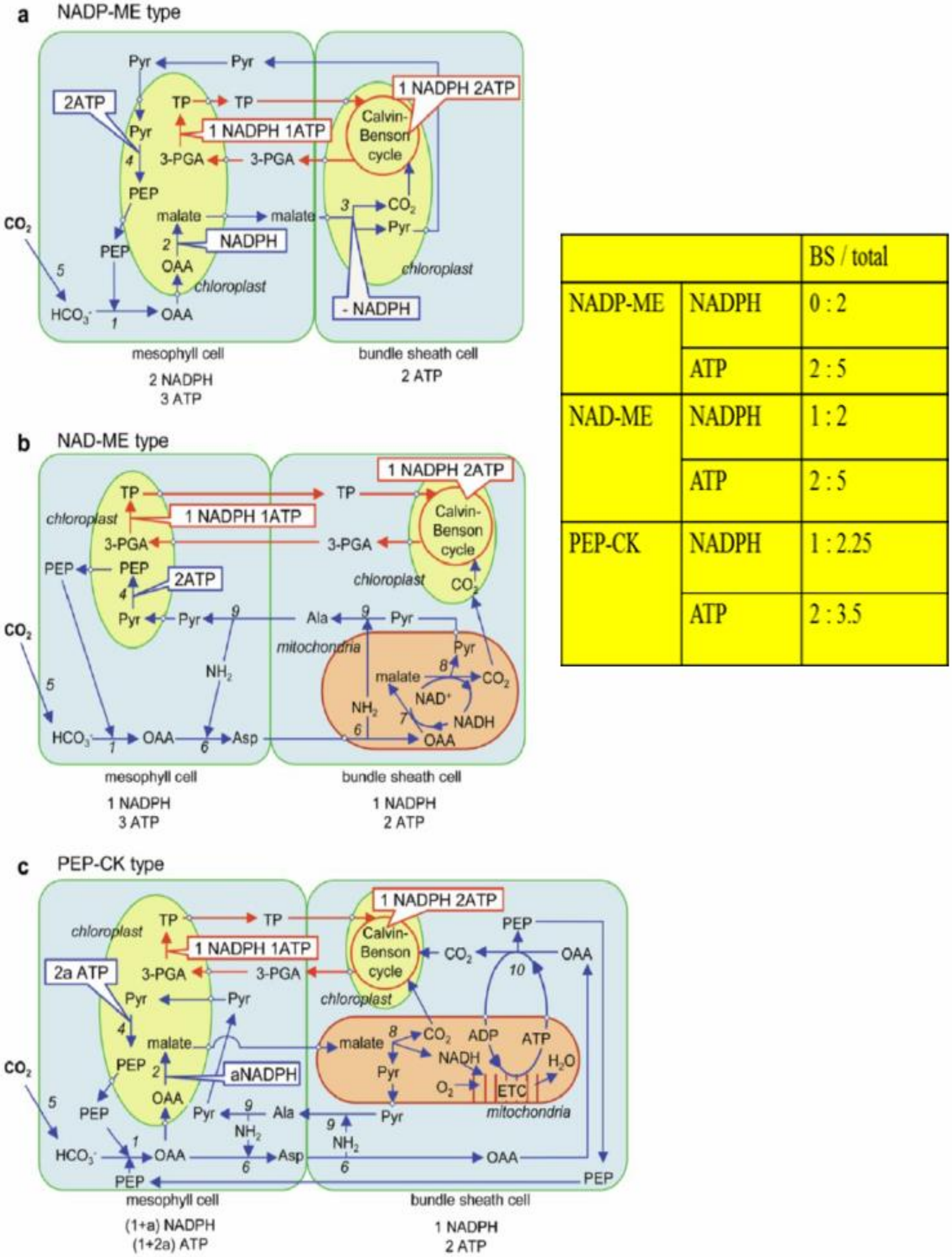

		BS / total
NADP-ME	NADPH	0 : 2
	ATP	2 : 5
NAD-ME	NADPH	1 : 2
	ATP	2 : 5
PEP-CK	NADPH	1 : 2.25
	ATP	2 : 3.5

Fig S1 The decarboxylation mechanism and minimum cell-type specific energy requirements (assuming 50% of the reduction of 3-PGA occurs in M and BS chloroplasts each) in three standard subtypes of C4 photosynthesis: (a) NADP-ME, (b) NAD-ME and (c) PEP-CK (drawn from Ishikawa et al. 2016 with permission). The table on the right summarises the minimum energy requirements in BS relative to the total.

Methods S1 The analytical model for cell-type specific electron transport

Here we describe the model on energy production, in a step-wise manner. First, basic model equations for ATP and NADPH production and quantum yield for CO_2 assimilation (Φ_{CO2}) are given for the case where only CO_2 fixation is considered, particularly for NADP-ME and NAD-ME subtypes. Special cases of a low ATP:NADPH requirement as occurring in the standard PEP-CK subtype as well as in the hypothetical "pure" PEP-CK type are then modelled. The model was further extended to account for the effects of photorespiration and alternative electron and ATP sinks on the cell energy budgets. How the model was adjusted to accommodate mixed decarboxylating types involving PEP-CK and to deal with other cases is also described. Finally, all model versions are summarised, and our model is compared with other existing models.

The model was presented in such a progressive manner with increasing complexity, for three reasons: (i) to reflect how the model was developed, (ii) for the purpose of clarifying: a complex version would have been hard to conceive and to understand without an earlier simpler version, and (iii) to have better insights about the importance of individual processes (for example, the minor contribution of photorespiration and alternative energy sinks indicated in the main text would not have been revealed if they were already included right at the beginning of the model).

1. Basic model for ATP and NADPH production

Both LET and CET may operate in M and BS cells (Table M1); so, total ATP production rate J_{atp} (see Table M2 for all symbol definitions) can be expressed as:

$$J_{atp} = [H_{LET}(J_{LET,M} + J_{LET,BS}) + H_{CET}(J_{CET,M} + J_{CET,BS})]/h \quad (1)$$

Table M1. Model symbols for indicating amounts of PSII and PSI for linear electron transport (LET) in bundle sheath cells (BS) and mesophyll cells (M), and amounts of PSI for cyclic electron transport (CET) in BS and M

	BS		M	
	PSII	PSI	PSII	PSI
LET	αT	$\frac{\Phi_{2LL}}{\Phi_{1LL}}\alpha T$	$(1-\alpha)T$	$\frac{\Phi_{2LL}}{\Phi_{1LL}}(1-\alpha)T$
CET		βC_x		$(1-\beta)C_x$

where in eqn (1), $J_{LET,M}$ and $J_{LET,BS}$ are the rate of LET in M and BS cells, respectively, and $J_{CET,M}$ and $J_{CET,BS}$ are the rate of CET in M and BS cells, respectively; h is the H^+ requirement per ATP synthesis (most likely h = 4, Yin & Struik 2012); H_{LET} and H_{CET} are the number of H^+ produced per electron transferred by LET and CET, respectively.

The number of H^+ per electron moving along LET or CET depends on the operation of the Q cycle such that $H_{LET} = 2 + f_Q$ and $H_{CET} = 1 + f_Q$ (Yin et al. 2004; where f_Q is the fraction of electrons at plastoquinone that follow the Q cycle). Since an obligatory Q cycle is generally accepted, 1 electron along LET and CET gives rise to 3 and 2 H^+, respectively. However, the stoichiometry for H_{CET} may depend on the pathway of CET (Kramer & Evans 2011; see later), but the quantitative analysis of C_4 quantum yield by Yin & Struik (2012) suggests that, most likely, H_{CET} = 2, arising from 1 + f_Q when the Q cycle is obligatorily operated (Yin et al. 2004).

NADPH production only depends on LET as:

$$J_{\text{nadph}} = 0.5(J_{\text{LET,M}} + J_{\text{LET,BS}}) \tag{2}$$

where 0.5 arises from the fact that 2 mol electrons are required to produce 1 mol NADPH.

The amounts of ATP and NADPH produced in BS cells are

$$J_{\text{atp,BS}} = (H_{\text{LET}} J_{\text{LET,BS}} + H_{\text{CET}} J_{\text{CET,BS}}) / h \tag{3}$$

$$J_{\text{nadph,BS}} = 0.5 J_{\text{LET,BS}} \tag{4}$$

So, the fraction of total ATP or NADPH that is produced in BS cells is:

$$f_{\text{atp,BS}} = J_{\text{atp,BS}} / J_{\text{atp}} \tag{5}$$

$$f_{\text{nadph,BS}} = J_{\text{nadph,BS}} / J_{\text{nadph}} \tag{6}$$

Both LET and CET depend on absorbed irradiance (I_{abs}). We consider low to moderate levels of light where photosynthesis is limited by electron transport, including limiting light conditions from which quantum yields for CO_2 assimilation can be measured. As stated in the main text, such light conditions would avoid any confounding effect of processes other than cellular energetics on our analysis. For LET, one needs to take into account the commonly observed relative difference in electron transport efficiency between PSI and PSII (Yin et al. 2004; Yin & Struik 2012); so, the electron transport rates under limiting light for all electron-transport types can be expressed as:

$$J_{\text{LET,M}} = \frac{\Phi_{2\text{LL}}}{1 + \Phi_{2\text{LL}} / \Phi_{1\text{LL}}} u(a_{\text{M}} I_{\text{inc}}) \tag{7}$$

$$J_{\text{LET,BS}} = \frac{\Phi_{2\text{LL}}}{1+\Phi_{2\text{LL}}/\Phi_{1\text{LL}}} v(a_{\text{BS}} I_{\text{inc}}) \quad (8)$$

$$J_{\text{CET,M}} = \Phi_{1\text{LL}}(1-u)(a_{\text{M}} I_{\text{inc}}) \quad (9)$$

$$J_{\text{CET,BS}} = \Phi_{1\text{LL}}(1-v)(a_{\text{BS}} I_{\text{inc}}) \quad (10)$$

where I_{inc} is the irradiance incident on the leaf, a_M and a_{BS} are the absorptance by M and BS chloroplasts, respectively [$I_{abs}=(a_M+a_{BS})I_{inc}$], u is the fraction of light for LET in M chloroplasts, v is the fraction of light for LET in BS chloroplasts, and Φ_{1LL} and Φ_{2LL} are the electron transport efficiencies under limiting light for PSI and PSII, respectively. In principle, our model applies to any light levels within the electron-transport limited range, by replacing Φ_{1LL} and Φ_{2LL} in eqns (7-10) by electron transport efficiencies of PSI and PSII at a given irradiance (Φ_1 and Φ_2), respectively. The efficiencies under limiting light are used here because we want to link these equations to calculate Φ_{CO2} (see later).

Mathematical derivations to solve intermediate parameters will be given later in Derivations A-C of this Supporting Information. Factors u and v will be analytically solved as a function of other parameters (Derivation A), based on total amounts of PSII per unit leaf area (T) and amounts of PSI per unit leaf area used for CET (C_x) in BS and M cells (Table M1). To solve u and v, it is necessary to analytically derive the solution for (i) the relative per unit photosystem absorptance between BS and M cells (k_{BS}/k_M), and (ii) the fraction of total PSI used for CET that is in BS cells (β). This is given in Derivation B, using the fraction of the whole-leaf PSI that lies in BS cells, f_{bsPSI}. That fraction can be experimentally measured (Ghannoum et al. 2005). In addition, to solve for k_{BS}/k_M and β, it is necessary to know the relative absorptance of irradiance between BS and M cells (a_{BS}/a_M). This is quantified in Derivation C using a simple model, the Beer-Lamberts law with the cumulative chlorophyll contents (Evans 1995), based on the schematic structure in Fig. 1.

The fraction of CET in the whole-leaf, in BS cells and in M cells would be:

$$f_{\text{CET}} = \frac{J_{\text{CET,M}} + J_{\text{CET,BS}}}{J_{\text{LET,M}} + J_{\text{LET,BS}} + J_{\text{CET,M}} + J_{\text{CET,BS}}} \quad (11)$$

$$f_{\text{CET,BS}} = J_{\text{CET,BS}} / (J_{\text{LET,BS}} + J_{\text{CET,BS}}) \quad (11a)$$

$$f_{\text{CET,M}} = J_{\text{CET,M}} / (J_{\text{LET,M}} + J_{\text{CET,M}}) \quad (11b)$$

Gross CO_2 assimilation rate, determined by NADPH and ATP supply to the C_3 cycle, is:

$$A_{g,nadph} = J_{nadph} / [2 + a(1+\phi)] \quad (12)$$

$$A_{g,atp} = J_{atp} / [3 + \varphi(1+\phi)] \quad (13)$$

where 2 and 3 are mol NADPH and ATP, respectively, required per C_3 cycle, *a* is additional mol NADPH required in the PEP-CK subtype for reducing OAA into malate (*a* has to be set to 0 when eqn 12 is applied for NADP-ME or NAD-ME subtypes), φ is mol ATP required per mol C_4 carboxylation (= 2 mol for NADP-ME and NAD-ME subtypes, and = 2*a* mol for the PEP-CK subtype), and ϕ is leakiness. The actual gross CO_2 assimilation rate (A_g) is co-limited by NADPH and ATP supply, as our model is formulated to ensure that

$$A_g = A_{g,nadph} = A_{g,atp} \quad (14a)$$

Quantum yield for CO_2 assimilation under limiting light (Φ_{CO2}) can be expressed as:

$$\Phi_{CO2} = A_g / [(a_M + a_{BS}) I_{inc}] \quad (14b)$$

2. Specific cases of low ATP:NADPH requirement as occurring in the standard PEP-CK subtype and in the "pure" PEP-CK type

Compared with NADP-ME and NAD-ME subtypes (which require 5:2 for the ATP:NADPH when there is no leakiness), the standard PEP-CK subtype has a different ratio of ATP:NADPH requirement. The minimum value is (3+2*a*) mol ATP and (2+*a*) mol NADPH per mol CO_2 assimilation, where a = 0.286 or 0.250 depending on whether ATP produced per oxidation of NADH in the respiratory electron transport (*n*) is 2.5 or 3.0 [$a = 1/(1+n)$, see Introduction and Fig. S1]. If leakage occurs, these requirements are somewhat higher. To run the model for the PEP-CK subtype, care needs to be taken to account for the higher rate of LET needed to meet a higher NADPH requirement. The NADPH requirement per CO_2 fixed is $2 + a(1+\phi)$; so, the required LET is $2[2 + a(1+\phi)]$, and the parameter *p* in eqn (A1) needs to be adjusted to:

$$p = 2[2 + a(1+\phi)](2 + f_Q)/h \quad (15)$$

where $(2+f_Q)/h$ is H_{LET}/h, ATP produced per electron transferred by LET (Yin et al. 2004). In case that $f_Q = 1$, eqn (15) predicts that $p = 3$ if $a = 0$, as has so far been the case applied to NADP-ME and NAD-ME subtypes. For the PEP-CK subtype where a = 0.286 or 0.25, *p* needs to be adjusted to 3.50 or 3.44 if leakiness ϕ is 0.16.

The extra amounts of LET to meet the extra NADPH requirement per CO_2 fixed, $2a(1+\phi)$, also produce ATP, which is $2a(1+\phi)(2 + f_Q)/h$. This would mean that if $f_Q = 1$ combined with

h = 4, this extra LET would meet ¾ of $2a(1+\phi)$, the extra ATP required for C_4 photosynthesis of the PEP-CK subtype, and the remaining ¼ of the extra ATP only requires a small amount of CET. To account for this, the denominator of the right side of eqn (A1), w, which is $\varphi(1+\phi)$ for NADP-ME and NAD-ME subtypes (where $\varphi = 2$), needs to be adjusted to the following for the PEP-CK subtype (where $\varphi = 2a$):

$$w = \varphi(1+\phi) - 2a(1+\phi)(2+f_Q)/h. \quad (16)$$

Such an adjustment would allow a balanced ATP and NADPH production, ensuring that Φ_{CO2} in terms of ATP requirements are equal to Φ_{CO2} in terms of NADPH requirements.

For the hypothetical "pure" PEP-CK type without using mitochondrial electron transport to provide ATP, the ATP to fuel PEP-CK has to come from chloroplastic electron transport. For this hypothetical type, eqn (15) and eqn (16) still apply, on the condition that a in eqns (15) and (16) is set to 0 (to accommodate that no NADPH from M chloroplasts is used for ATP production from NADH oxidation in BS mitochondria) and φ (mol ATP required per mol C_4 carboxylation) is adjusted to 1 (1 mol ATP required for PEP-CK to directly decarboxylate 1 mol OAA into CO_2 and PEP). So, the minimum value is (3+1) mol ATP and 2 mol NADPH per mol CO_2 assimilation, and its ATP:NADPH ratio is 4:2, higher than that for the standard PEP-CK subtype, but lower than that for the two ME subtypes. If considering leakiness, the ATP:NADPH ratio for the "pure" PEP-CK type is $(4+\phi)$:2.

3. Accounting for photorespiration and alternative electron and ATP sinks

In this section, our model is extended to quantify the effects of photorespiration and other electron- and ATP-consuming processes on the energy budget and quantum yield. Nitrate reduction and starch synthesis are considered as two major alternative processes utilising chloroplastic electrons and ATP. The Mehler reaction is not considered here as this reaction is negligible under conditions where photosynthesis is limited by electron transport. Sucrose synthesis, which occurs in the M cytosol, will not be considered either as it consumes no additional NADPH or ATP (Amthor 2010). The malate valve may act on the NADPH and ATP balance (Kramer & Evans 2011), but reductants exported from chloroplasts by this valve may be used for nitrate reduction; so any operation of this mechanism is lumped to nitrate reduction, as energy costs for the latter process are more quantifiable. Note that fractions of the whole-chain

electrons consumed by all these alternative processes are lumped into the term f_{pseudo} for the "pseudocyclic" form in our previous whole-leaf model (Yin & Struik 2012).

Per mol RuBP oxygenation 2 mol NADPH and 3.5 mol ATP are consumed, 0.5 mol ATP more than per mol RuBP carboxylation (Farquhar et al. 1980). Per mol nitrate reduction 10 mol electrons and 1 mol ATP are consumed (Noctor & Foyer 1998). If starch is considered as the end product of photosynthesis, there is a cost of 2/12 (= 0.167) mol ATP per mol CO_2 for polymerising one mol hexose into starch (Noctor & Foyer 1998; Amthor 2010). So, parameter p in eqn (A1), equivalent to eqn (15) above, needs to be further adjusted to:

$$p = \{2[2 + a(1+\phi) + 2\upsilon_{o/c}] + 10\upsilon_{n/c}\}(2 + f_Q)/h \quad (17)$$

Parameter w in eqn (A1), equivalent to eqn (16) above, needs to be adjusted to:

$$w = \varphi(1+\phi) - [2a(1+\phi) + 10\upsilon_{n/c}](2 + f_Q)/h + 0.5\upsilon_{o/c} + \upsilon_{n/c} + c_{starch}(1 - 0.5\upsilon_{o/c} - \upsilon_{r/c}) \quad (18)$$

where $\upsilon_{o/c}$ is the RuBP oxygenation to RuBP carboxylation ratio, $\upsilon_{n/c}$ is the nitrate reduction to RuBP carboxylation ratio, $\upsilon_{r/c}$ is the day respiration to RuBP carboxylation ratio, and c_{starch} is the ATP cost for starch synthesis (= 0.167). Gross CO_2 assimilation rate, equivalent to eqns (12) and (13), also need to be adjusted:

$$A_{g,nadph} = J_{nadph}/[2 + a(1+\phi) + 2\upsilon_{o/c} + 5\upsilon_{n/c}] \quad (19)$$

$$A_{g,atp} = J_{atp}/[3 + \varphi(1+\phi) + 3.5\upsilon_{o/c} + 1.0\upsilon_{n/c} + c_{starch}(1 - 0.5\upsilon_{o/c} - \upsilon_{r/c})] \quad (20)$$

Equations (18) and (20) assume (i) that the rate of starch synthesis (in μmol CO_2 m^{-2} s^{-1}) is equal to ($V_c - 0.5V_o - R_d$), where V_c, V_o and R_d correspond to rates of RuBP carboxylation, RuBP oxygenation and day respiration, respectively, and (ii) that per mol RuBP oxygenation 0.5 mol CO_2 is released. All these equations ensure again that the whole-leaf Φ_{CO2} in terms of ATP requirement and the whole-leaf Φ_{CO2} in terms of NADPH requirement are equal.

4. Adjusting the model to accommodate energy production in mixed types involving PEP-CK

Unlike the mixed "NADP-ME + aspartate-malate" mechanism where the total whole-leaf energy requirement per CO_2 fixed stays the same as that of the NADP-ME pathway, the total ATP requirement in the mixed types having PEP-CK changes compared with the primary NADP-ME or NAD-ME pathway (Table 4). However, only parameter φ (mol ATP required per mol C_4 carboxylation) in eqns (18) and (20) needs to be adjusted from 2 for NAD(P)-ME subtypes to $(1+\eta)$ for the mixed types, where η be the fraction of OAA following the primary NADP-ME (or

NAD-ME) route and the remaining $(1-\eta)$ be the fraction following the secondary "PEP-CK" mechanism. Because the model for calculating the fractions of NADPH or ATP produced in BS cells and the model for the fraction of ATP required in BS cells both need parameter η, this would need an iterative approach to solve η. However, using a range of pre-set values for η showed that the calculated fractions of NADPH or ATP produced in BS cells changed little with η, meaning that the fractions of NADPH or ATP produced in BS cells were largely determined by other parameters such as α, f_{bsCHL} and f_{bsPSI} (see Table M2 for their definition). This insensitivity simplifies the analysis for the mixed type involving PEP-CK.

5. Considering other possible values of h, f_Q and H_{CET}

Stoichiometric coefficients related to ATP production are uncertain. For example, the H^+:ATP ratio (*h*) is often also believed to be 14/3 or 4.67 (Kramer & Evans 2011), based on the structural data that the H^+-driven turbine of the chloroplast ATPase has 14 subunits. If h = 4.67, then the ATP:NADPH ratio generated by LET in combination with the Q cycle is lower than the required 1.5 for the C_3 cycle, and the shortfall in ATP must come from a higher fraction for CET. A similar case requiring more CET is when the Q cycle is partially operated ($f_Q < 1$) while h is 4. In either case, parameter w in eqn (18) then needs to be completed with another term:

$$w = \varphi(1+\phi) - [2a(1+\phi) + 10\upsilon_{n/c}](2+f_Q)/h + 0.5\upsilon_{o/c} + \upsilon_{n/c} + c_{starch}(1-0.5\upsilon_{o/c} - \upsilon_{r/c}) + [3-4(2+f_Q)/h](1+\upsilon_{o/c}) \quad (21)$$

where 3 is the number of ATP, and 4 is the number of linear electrons required to produce 2 mol NADPH, required for 1 mol CO_2 assimilation by the Calvin cycle, so the whole term $[3-4(2+f_Q)/h](1+\upsilon_{o/c})$ is the shortfall of ATP required by both the Calvin cycle and the photorespiratory cycle per mol CO_2 assimilated if $h > 4$ or if $f_Q < 1$. Actually this extension also applies to the case where $h < 4$, like in earlier days when h was considered to be 3 (Furbank et al. 1990). In such a case, there may be an overproduction of ATP by LET so that a lower CET would be required. If h is as low as 3, then the value of f_Q may also need to be lowered (i.e., a zero or partial Q cycle), especially for the PEP-CK types, to avoid too much overproduction of ATP by LET. Otherwise, our model will predict an unrealistic value of parameters u and v, which would suggest, as discussed in the main text, an impossibility of certain (sets of) input parameter values.

Another uncertain parameter is H_{CET}. As stated in the main text, H_{CET} could become higher than 1+ f_Q, our default expression for H_{CET}, because two extra H^+ are generated if CET runs in the NAD(P)H dehydrogenase (NDH)-dependent pathway (Kramer & Evans 2011). Our model can accommodate this possibility if H_{CET} is changed to be expressed as $1 + f_Q + 2f_{NDH}$, where f_{NDH} is the fraction of the whole-leaf CET that follows the NDH pathway.

Our model ensures an equal whole-leaf Φ_{CO2} in terms of ATP and NADPH requirement for all these possibilities. However, these possibilities are no longer further discussed in the main text and elsewhere of the Supporting Information, where we stay with the most likely, simple scenario that $h = 4$, $f_Q = 1$, $H_{CET} = 1+f_Q$, and $H_{LET} = 2+f_Q$ (Yin & Struik 2012).

6. Summary of various model versions

Model was presented above with seemingly increasing complexity to show a step-wise approach to its development. However, the variation of model versions lies in the expressions of only *p*, *w*, $A_{g,nadph}$ and $A_{g,atp}$. Eqns (17, 19-21) for them still hold for all various C_4 types, but only two parameters, *a* and φ, in these equations have to be (sub)type-specific:

$$a = \begin{cases} 1/(1+n) & \text{for standard PEP-CK subtype} \\ 0 & \text{for all other types} \end{cases} \tag{22}$$

$$\varphi = \begin{cases} 2 & \text{for two ME subtypes, and "NADP-ME+aspartate-malate" mixed type} \\ 2/(1+n) & \text{for standard PEP-CK subtype} \\ 1 & \text{for "pure" PEP-CK subtype} \\ 1+\eta & \text{for "NAD(P)-ME+PEP-CK" mixed types} \end{cases} \tag{23}$$

where *n* is relevant to the standard PEP-CK subtype, referring to mol ATP produced per oxidation of NADH in mitochondrial electron transport chain ($n = 2.5$ or 3; see the main text). For the model version without considering photorespiration and alternative electron and ATP sinks, $\upsilon_{o/c}$, $\upsilon_{n/c}$, $\upsilon_{r/c}$ and c_{starch} in eqns (17-21) just need to be set to 0. Algorithms and solutions in Derivations A-C stay the same for all model versions.

7. Comparison of our model with existing C_4 models

Several models have been published for C_4 cell-type specific processes (e.g. Wang et al. 2014), thereby going beyond those classical models (Farquhar 1983; Furbank et al. 1990; von Caemmerer & Furbank 1999) and a recent model of Yin & Struik (2012) for whole-leaf C_4

photosynthesis. In particular, papers of Bellasio & Griffiths (2014), Bellasio & Lundgren (2016) and Bellasio (2017) also address the bioenergetics detailed in our model. Therefore, it is necessary to compare our model with these existing models.

As stated in Introduction, the model of Wang et al. (2014) for numerical simulation of various C_4 subtypes or mixed types does not incorporate CET; so the difference between this numerical simulation model and our analytical model is obvious.

The model of Bellasio & Griffiths (2014) did incorporate CET as well as detailed stoichiometric algorithms for cell-type specific metabolic processes. However, both ATP production and metabolic processes were tailored for maize, a consummate NADP-ME species. Its submodel for ATP supply, considering light penetration in dependence of anatomical traits, was extended to analyse whether sufficient ATP could be produced in BS cells in the context of the evolutionary continuum from C_3 to C_4 (Bellasio & Lundgren 2016). The model of Bellasio & Griffiths (2014) and Bellasio & Lundgren (2016) mainly quantified the relative ATP production in the different cell types, expressed as BS:M ratio ($J_{\mathrm{atp,BS}}/J_{\mathrm{atp,M}}$).

In contrast, our model presented here suits for predicting cell-type specific NADPH as well as ATP production for all various C_4 subtypes or mixed types. From our model eqns (1) and (3), $J_{\mathrm{atp,BS}}/J_{\mathrm{atp,M}}$ can be formulated:

$$\frac{J_{\mathrm{atp,BS}}}{J_{\mathrm{atp,M}}} = \frac{(H_{\mathrm{LET}} J_{\mathrm{LET,BS}} + H_{\mathrm{CET}} J_{\mathrm{CET,BS}})/h}{(H_{\mathrm{LET}} J_{\mathrm{LET,M}} + H_{\mathrm{CET}} J_{\mathrm{CET,M}})/h} \tag{24}$$

Substituting eqns (7-10) into eqn (24) gives:

$$\frac{J_{\mathrm{atp,BS}}}{J_{\mathrm{atp,M}}} = \frac{\dfrac{H_{\mathrm{LET}}}{h} \dfrac{\Phi_{\mathrm{2LL}}}{1+\Phi_{\mathrm{2LL}}/\Phi_{\mathrm{1LL}}} v + \dfrac{H_{\mathrm{CET}}}{h} \Phi_{\mathrm{1LL}}(1-v)}{\dfrac{H_{\mathrm{LET}}}{h} \dfrac{\Phi_{\mathrm{2LL}}}{1+\Phi_{\mathrm{2LL}}/\Phi_{\mathrm{1LL}}} u + \dfrac{H_{\mathrm{CET}}}{h} \Phi_{\mathrm{1LL}}(1-u)} \frac{a_{\mathrm{BS}}}{a_{\mathrm{M}}} \tag{25}$$

In eqn (25), the lumped coefficient in front of u and v is the efficiency of LET in converting absorbed light into ATP (denoted as η_{LET}), and the lumped coefficient in front of $(1-u)$ and $(1-v)$ represents the efficiency of CET in converting absorbed light into ATP (denoted as η_{CET}). Then eqn (25) becomes:

$$\frac{J_{\mathrm{atp,BS}}}{J_{\mathrm{atp,M}}} = \frac{\eta_{\mathrm{LET}} v + \eta_{\mathrm{CET}}(1-v)}{\eta_{\mathrm{LET}} u + \eta_{\mathrm{CET}}(1-u)} \frac{a_{\mathrm{BS}}}{a_{\mathrm{M}}} \tag{26}$$

If $u = 1$, eqn (26) becomes:

$$\frac{J_{\text{atp,BS}}}{J_{\text{atp,M}}} = \left[1 + (1-v)\left(\frac{\eta_{\text{CET}}}{\eta_{\text{LET}}} - 1\right)\right]\frac{a_{\text{BS}}}{a_{\text{M}}} \tag{27}$$

This is exactly eqn (2) of Bellasio & Lundgren (2016). It is clear that their model is *a special case* of our model when absorbed light by M cells is used only for LET (u = 1). As shown in the main text, this assumption holds approximately only for the NADP-ME subtype and does not hold for NAD-ME and PEP-CK subtypes. In the model of Bellasio & Griffiths (2014), $J_{\text{atp,BS}}/J_{\text{atp,M}}$ was further simplified to $2a_{\text{BS}}/a_{\text{M}}$ (their eqn 3), resulting from additional assumptions that v = 0 and $\eta_{\text{CET}}/\eta_{\text{LET}}$ = 2. Again the assumption that v = 0 (absorbed light in BS cells is used only for CET) holds approximately only for the NADP-ME subtype. From our discussion in the main text, it is also hard to exactly reconcile the assumption that $\eta_{\text{CET}}/\eta_{\text{LET}}$ = 2.

Instead of fixing them to approximate values, parameters u, v, and β (the fraction of PSI used for CET) in our model were solved analytically from current understanding of the most likely stoichiometry of C_4 physiology (see below for Derivations A and B), conditional on experimentally measurable parameters such as [CHL], f_{bsCHL} and f_{bsPSI} (Table 2). The algorithms in these derivations were carefully formulated to simultaneously account for (i) energy lost due to leakiness in addition to alternative electron and ATP sinks, and (ii) the balanced production of NADPH and ATP that co-limit the photosynthetic rate. Neither of the latter two aspects was considered explicitly in the model of Bellasio and colleagues. Because of the coherent analytical algorithms conditional on C_4 physiology and some input parameter values, our model allows the solving of the physiologically plausible range of the variation in other parameters as shown for α (the fraction of PSII that is in BS cells) in various C_4 types (Tables 3, 5 and 6 in the main text). This feature of our model in combining C_4 physiology and analytical mathematics also allows us, as shown in the main text, to identify knowledge gaps that could be used to design new experimental studies.

The model of Bellasio & Griffiths (2014) has detailed cell-type specific stoichiometries for maize metabolic processes that consume NADPH and ATP, and this was extended for various photosynthetic types C_3, C_2, C_2+C_4, and C_4 including the three subtypes and mixed types (Bellasio 2017). Our algorithms for these stoichiometries on NADPH and ATP demands by M and BS cells in various C_4 types are in an intermediate detail between those of Bellasio and colleagues and the classical C_4 model, and are summarised as a table, Table 4. This allowed us to analytically solve the required fraction of 3-PGA reduction in each cell type and the fraction for

the primary and secondary decarboxylation (see the main text). Furthermore, Bellasio & Griffiths (2014) and Bellasio & Lundgren (2016) numerically modelled the relative BS/M light capture (a_{BS}/a_M) from underlying absorption and scattering coefficients, thereby generating light penetration profiles dependent on light spectrum (Bellasio & Griffiths 2014). In comparison, we used a simpler descriptive approach using light extinction coefficient k based on the experimental observation of Evans (1995) (Derivation C). A sensitivity analysis of our model with respect to the value of k will be given in Notes S4 (see later).

Derivation A Deriving equations that express v and u as a function of other parameters

Let T be the total amount of PSII per unit leaf area in BS and M cells; then αT will be the amount of PSII in BS cells (where α is the fraction of PSII in BS cells) and $(1-\alpha)T$ will be the amount of PSII in M cells (Table M1). Note that α has the same meaning as used in the classical C_4-photosynthesis model of von Caemmerer & Furbank (1999).

To account for the difference in the electron transport efficiency between PSI and PSII (Yin et al. 2004), the amount of PSI in BS cells has to be $(\Phi_{2LL}/\Phi_{1LL})\alpha T$ to enable an equal electron transport flux passing through PSI and PSII for LET; similarly the amount of PSI in M cells has to be $(\Phi_{2LL}/\Phi_{1LL})(1-\alpha)T$ (Table M1).

Let the total amount of PSI per unit leaf area used for CET be C_x; its fraction in BS is β, and the remaining fraction, $1-\beta$, is in M (Table M1). We now need to solve C_x, based on ATP requirement for C_4 physiology. ATP produced from total LET should be $\frac{H_{\mathrm{LET}}}{h}[k_{BS}\alpha T+k_M(1-\alpha)T]\Phi_{2LL}$ (where k_M and k_{BS} are mol photons absorbed per unit photosystem in M and BS chloroplasts, respectively), and ATP produced from CET should be $\frac{H_{\mathrm{CET}}}{h}[k_{BS}\beta C_x+k_M(1-\beta)C_x]\Phi_{1LL}$. It is hard to determine the absolute values of k_M and k_{BS}, but only their ratio is relevant in calculating factors v and u, as shown below. The relative k_M and k_{BS} ratio of a whole-leaf depends on the relative Chl content per photosystem in M and BS cells. Also β cannot be measured although it must depend on the relative area of BS and M tissues and the density of PSI in each cell type. A method to solve β and k_{BS}/k_M will be given in Derivation B.

It is recognised that NADPH and ATP and their ratio generated from LET if h = 4 and if the Q cycle is fully operated exactly match the requirement for the C_3 cycle. Thus, additional ATP requirement for the C_4 cycle must be satisfied from CET which does not generate NADPH. Let p

be mol ATP required by the C_3 cycle that is satisfied from LET, and let *w* be mol ATP requirement by the C_4 cycle from chloroplastic electron transport, per mol CO_2 fixation, then the following can be written:

$$\frac{\frac{H_{\mathrm{LET}}}{h}[k_{\mathrm{BS}}\alpha T+k_{\mathrm{M}}(1-\alpha)T]\Phi_{2\mathrm{LL}}}{\frac{H_{\mathrm{CET}}}{h}[k_{\mathrm{BS}}\beta C_{\mathrm{x}}+k_{\mathrm{M}}(1-\beta)C_{\mathrm{x}}]\Phi_{1\mathrm{LL}}}=\frac{p}{w} \tag{A1}$$

For NADP-ME and NAD-ME subtypes, *w* should be equal to $\varphi(1+\phi)$, where φ is mol ATP required for PEP regeneration and ϕ is leakiness (see above for the discussion on *w* for the PEP-CK subtype). This results in:

$$C_{\mathrm{x}}=\frac{H_{\mathrm{LET}}w\Phi_{2\mathrm{LL}}[k_{\mathrm{BS}}\alpha+k_{\mathrm{M}}(1-\alpha)]}{H_{\mathrm{CET}}p\Phi_{1\mathrm{LL}}[k_{\mathrm{BS}}\beta+k_{\mathrm{M}}(1-\beta)]}T \tag{A2}$$

The total amount of light absorbed by BS ($I_{\mathrm{abs,BS}}$) can be written, based on Table M1, as:

$$\begin{aligned}I_{\mathrm{abs,BS}}&=k_{\mathrm{BS}}\left(\alpha T+\frac{\Phi_{2\mathrm{LL}}}{\Phi_{1\mathrm{LL}}}\alpha T+\beta C_{\mathrm{x}}\right)\\&=k_{\mathrm{BS}}T\left\{\alpha+\frac{\Phi_{2\mathrm{LL}}}{\Phi_{1\mathrm{LL}}}\alpha+\beta\frac{H_{\mathrm{LET}}w\Phi_{2\mathrm{LL}}[k_{\mathrm{BS}}\alpha+k_{\mathrm{M}}(1-\alpha)]}{H_{\mathrm{CET}}p\Phi_{1\mathrm{LL}}[k_{\mathrm{BS}}\beta+k_{\mathrm{M}}(1-\beta)]}\right\}\end{aligned} \tag{A3}$$

The amount of light absorbed by BS that is used for LET ($I_{\mathrm{abs,BS,LET}}$) can be written, based on Table M1, as:

$$I_{\mathrm{abs,BS,LET}}=k_{\mathrm{BS}}\left(\alpha T+\frac{\Phi_{2\mathrm{LL}}}{\Phi_{1\mathrm{LL}}}\alpha T\right) \tag{A4}$$

The parameter *v*, by definition, is the ratio of $I_{\mathrm{abs,BS,LET}}$ to $I_{\mathrm{abs,BS}}$, which can be solved from the above two equations as:

$$v=\frac{1+\frac{\Phi_{2\mathrm{LL}}}{\Phi_{1\mathrm{LL}}}}{1+\frac{\Phi_{2\mathrm{LL}}}{\Phi_{1\mathrm{LL}}}\left\{1+\frac{H_{\mathrm{LET}}w\beta[(\alpha k_{\mathrm{BS}}/k_{\mathrm{M}})+(1-\alpha)]}{H_{\mathrm{CET}}p\alpha[(\beta k_{\mathrm{BS}}/k_{\mathrm{M}})+(1-\beta)]}\right\}} \tag{A5}$$

Similar logic can be used to solve for factor *u*. The total amount of light absorbed by M ($I_{\mathrm{abs,M}}$) can be written, based on Table M1, as:

$$\begin{aligned}I_{\mathrm{abs,M}}&=k_{\mathrm{M}}\left[(1-\alpha)T+\frac{\Phi_{2\mathrm{LL}}}{\Phi_{1\mathrm{LL}}}(1-\alpha)T+(1-\beta)C_{\mathrm{x}}\right]\\&=k_{\mathrm{M}}T\left\{1-\alpha+\frac{\Phi_{2\mathrm{LL}}}{\Phi_{1\mathrm{LL}}}(1-\alpha)+(1-\beta)\frac{H_{\mathrm{LET}}w\Phi_{2\mathrm{LL}}[k_{\mathrm{BS}}\alpha+k_{\mathrm{M}}(1-\alpha)]}{H_{\mathrm{CET}}p\Phi_{1\mathrm{LL}}[k_{\mathrm{BS}}\beta+k_{\mathrm{M}}(1-\beta)]}\right\}\end{aligned} \tag{A6}$$

The amount of light absorbed by M that is used for LET ($I_{\mathrm{abs,M,LET}}$) can be written, based on Table M1, as:

$$I_{\mathrm{abs,M,LET}}=k_{\mathrm{M}}\left[(1-\alpha)T+\frac{\Phi_{2\mathrm{LL}}}{\Phi_{1\mathrm{LL}}}(1-\alpha)T\right] \tag{A7}$$

The parameter *u*, by definition, is the ratio of $I_{\mathrm{abs,M,LET}}$ to $I_{\mathrm{abs,M}}$, which can be solved from the above two equations as:

$$u = \frac{1+\frac{\Phi_{2LL}}{\Phi_{1LL}}}{1+\frac{\Phi_{2LL}}{\Phi_{1LL}}\left\{1+\frac{H_{LET}w(1-\beta)[(\alpha k_{BS}/k_M)+(1-\alpha)]}{H_{CET}p(1-\alpha)[(\beta k_{BS}/k_M)+(1-\beta)]}\right\}} \tag{A8}$$

Derivation B   Deriving equations to solve for β and k_{BS}/k_M

Although parameter β (the fraction of C_x in BS cells) cannot be measured directly, the fraction of the whole-leaf PSI that lies in BS cells, f_{bsPSI}, can be experimentally measured (Ghannoum et al. 2005; Majeran et al. 2005). f_{bsPSI} can be written, by definition and according to Table M1, as:

$$f_{bsPSI} = \frac{PSI_{BS}}{PSI} = \frac{(\Phi_{2LL}/\Phi_{1LL})\alpha T + \beta C_x}{(\Phi_{2LL}/\Phi_{1LL})T + C_x} \tag{B1}$$

From this, C_x could be solved as:

$$C_x = \frac{\Phi_{2LL}(f_{bsPSI} - \alpha)T}{\Phi_{1LL}(\beta - f_{bsPSI})} \tag{B2}$$

According to the definitions and the information in Table M1, k_{BS} and k_M can be written as:

$$k_{BS} = \frac{I_{abs,BS}}{PS_{BS}} = \frac{I_{abs,BS}}{\alpha T + \frac{\Phi_{2LL}}{\Phi_{1LL}}\alpha T + \beta C_x} = \frac{I_{abs,BS}}{\left[\alpha + \frac{\Phi_{2LL}}{\Phi_{1LL}}\alpha + \frac{\Phi_{2LL}\beta(f_{bsPSI} - \alpha)}{\Phi_{1LL}(\beta - f_{bsPSI})}\right]T} \tag{B3}$$

$$k_M = \frac{I_{abs,M}}{PS_M} = \frac{I_{abs,M}}{(1-\alpha)T + \frac{\Phi_{2LL}}{\Phi_{1LL}}(1-\alpha)T + (1-\beta)C_x} = \frac{I_{abs,M}}{\left[(1-\alpha) + \frac{\Phi_{2LL}}{\Phi_{1LL}}(1-\alpha) + \frac{\Phi_{2LL}(1-\beta)(f_{bsPSI} - \alpha)}{\Phi_{1LL}(\beta - f_{bsPSI})}\right]T} \tag{B4}$$

The ratio of the two can be written:

$$\frac{k_{BS}}{k_M} = \frac{I_{abs,BS}}{I_{abs,M}} \frac{(1-\alpha) + \frac{\Phi_{2LL}}{\Phi_{1LL}}(1-\alpha) + \frac{\Phi_{2LL}(1-\beta)(f_{bsPSI} - \alpha)}{\Phi_{1LL}(\beta - f_{bsPSI})}}{\alpha + \frac{\Phi_{2LL}}{\Phi_{1LL}}\alpha + \frac{\Phi_{2LL}\beta(f_{bsPSI} - \alpha)}{\Phi_{1LL}(\beta - f_{bsPSI})}} \tag{B5}$$

This equation can be re-formulated as:

$$\frac{k_{BS}}{k_M} = \frac{b\beta + c}{d\beta + e} \tag{B6}$$

where $b = \frac{I_{abs,BS}}{I_{abs,M}}\left[(1-\alpha)\left(1+\frac{\Phi_{2LL}}{\Phi_{1LL}}\right) - (f_{bsPSI} - \alpha)\frac{\Phi_{2LL}}{\Phi_{1LL}}\right]$

$$c = \frac{I_{abs,BS}}{I_{abs,M}}\left[(f_{bsPSI} - \alpha)\frac{\Phi_{2LL}}{\Phi_{1LL}} - (1-\alpha)\left(1+\frac{\Phi_{2LL}}{\Phi_{1LL}}\right)f_{bsPSI}\right]$$

$$d = \alpha + \frac{\Phi_{2LL}}{\Phi_{1LL}} f_{bsPSI}$$

$$e = -\alpha f_{bsPSI}\left(1 + \frac{\Phi_{2LL}}{\Phi_{1LL}}\right)$$

The $I_{abs,BS}/I_{abs,M}$ ratio required for calculating b and c will be given in Derivation C.

There are two equations, eqn (A2) and eqn (B2), calculating C_x, and the two must be equal, that is:

$$\frac{H_{LET}\, w \Phi_{2LL}}{H_{CET}\, p \Phi_{1LL}} \frac{(k_{BS}/k_M)\alpha + (1-\alpha)}{(k_{BS}/k_M)\beta + (1-\beta)} = \frac{\Phi_{2LL}(f_{bsPSI} - \alpha)}{\Phi_{1LL}(\beta - f_{bsPSI})} \quad \text{(B7)}$$

Solving for k_{BS}/k_M and re-arranging all the terms give:

$$\frac{k_{BS}}{k_M} = \frac{f\beta + g}{h\beta + i} \quad \text{(B8)}$$

where $f = -\left[(f_{bsPSI} - \alpha)\frac{H_{CET}\, p}{H_{LET}\, w} + (1-\alpha)\right]$

$$g = (f_{bsPSI} - \alpha)\frac{H_{CET}\, p}{H_{LET}\, w} + (1-\alpha) f_{bsPSI}$$

$$i = \alpha - (f_{bsPSI} - \alpha)\frac{H_{CET}\, p}{H_{LET}\, w}$$

$$j = -\alpha f_{bsPSI}$$

The two k_{BS}/k_M, given by eqn (B6) and eqn (B8), must be equal, that is:

$$\frac{k_{BS}}{k_M} = \frac{b\beta + c}{d\beta + e} = \frac{f\beta + g}{i\beta + j} \quad \text{(B9)}$$

This gives the solution to β:

$$\beta = \begin{cases} \dfrac{-B + \sqrt{B^2 - 4AC}}{2A} & \text{if } \alpha < f_{bsPSI} \\ \dfrac{-B - \sqrt{B^2 - 4AC}}{2A} & \text{if } \alpha > f_{bsPSI} \end{cases} \quad \text{(B10)}$$

where $A = bi - df$, $B = bj + ci - dg - ef$, and $C = cj - eg$. In eqn (B10), the matching of $\alpha < f_{bsPSI}$ or $\alpha < f_{bsPSI}$ with the two solutions was determined from the fact that the C_x/T ratio has to be positive. According to eqn (B2), the positive C_x/T ratio requires that β has to be > f_{bsPSI} if $\alpha < f_{bsPSI}$ or β has to be < f_{bsPSI} if $\alpha > f_{bsPSI}$. Calculations show that within a physiologically relevant

range (i.e., u, v, and β are all within 0 and 1), solution 1 is always > f_{bsPSI} if $\alpha < f_{\mathrm{bsPSI}}$, solution 2 is always < f_{bsPSI} if $\alpha > f_{\mathrm{bsPSI}}$, and the two solutions are both equal to f_{bsPSI} if $\alpha = f_{\mathrm{bsPSI}}$. Because the denominator of eqn (B2) is zero if $\beta = f_{\mathrm{bsPSI}}$, it follows that α cannot equal f_{bsPSI}. Indeed, experimental data in Table 2 of the main text showed that $\alpha < f_{\mathrm{bsPSI}}$ in two NADP-ME and two NAD-ME species.

After β is solved, $k_{\mathrm{BS}}/k_{\mathrm{M}}$ can be solved from either eqn (B6) or eqn (B8). Then the C_{x}/T ratio can be calculated, and a whole-leaf PSI:PSII ratio can be calculated thereof (Table M1):

$$\frac{PSI}{PSII} = \frac{\Phi_{2\mathrm{LL}}}{\Phi_{1\mathrm{LL}}} + C_{\mathrm{x}} / T \tag{B11}$$

Or substituting eqn (B2) into the above equation gives:

$$\frac{PSI}{PSII} = \frac{\Phi_{2\mathrm{LL}}}{\Phi_{1\mathrm{LL}}}\left(1 + \frac{f_{\mathrm{bsPSI}} - \alpha}{\beta - f_{\mathrm{bsPSI}}}\right) \tag{B12}$$

Derivation C   Calculating a_M, a_{BS} and their ratio

Vertical light-absorption profile inside a leaf can be modelled at a different level of sophistication, and it is modelled here in a simplest possible way, based on the observation that the light profile obeys the Beer-Lamberts law with the cumulative chlorophyll contents (Evans 1995). Because this part of our model is independent of other parts, the algorithms could be replaced with more sophisticated ones if such sophisticated algorithms are deemed necessary to meet different goals of modelling analysis.

Often the fraction of the whole-leaf chlorophyll in BS cells, f_{bsCHL}, can be experimentally measured (Ghannoum et al. 2005). It is therefore possible to derive the $a_{\mathrm{BS}}/a_{\mathrm{M}}$ ratio, in relation to the anatomical distribution of M and BS cells (Fig. 1). The chlorophyll content in each of the BS, M1, M2 and M3 sections can be easily calculated from whole-leaf chlorophyll content, f_{bsCHL}, and schematic anatomical parameters as described in Fig. 1.

Assuming an absence of absorption by non-photosynthetic pigments, light absorption by M1, M2, BS and M3 can be formulated according to the Beer-Lambert law:

$$I_{\mathrm{abs,M1}} = mI_{\mathrm{inc}}(1 - e^{-k \cdot CHL_{\mathrm{M1}}}) \tag{C1}$$

$$I_{\mathrm{abs,M2}} = (1-m)I_{\mathrm{inc}}(1-e^{-k\cdot CHL_{\mathrm{M2}}}) \quad \text{(C2)}$$

$$\begin{aligned} I_{\mathrm{abs,BS}} &= (1-m)I_{\mathrm{inc}}(1-e^{-k\cdot(CHL_{\mathrm{M2}}+CHL_{\mathrm{BS}})}) - (1-m)I_{\mathrm{inc}}(1-e^{-k\cdot CHL_{\mathrm{M2}}}) \\ &= (1-m)I_{\mathrm{inc}}e^{-k\cdot CHL_{\mathrm{M2}}}(1-e^{-k\cdot CHL_{\mathrm{BS}}}) \end{aligned} \quad \text{(C3)}$$

$$\begin{aligned} I_{\mathrm{abs,M3}} &= (1-m)I_{\mathrm{inc}}(1-e^{-k\cdot(CHL_{\mathrm{M2}}+CHL_{\mathrm{BS}}+CHL_{\mathrm{M3}})}) - (1-m)I_{\mathrm{inc}}(1-e^{-k\cdot(CHL_{\mathrm{M2}}+CHL_{\mathrm{BS}})}) \\ &= (1-m)I_{\mathrm{inc}}e^{-k\cdot(CHL_{\mathrm{M2}}+CHL_{\mathrm{BS}})}(1-e^{-k\cdot CHL_{\mathrm{M3}}}) \end{aligned} \quad \text{(C4)}$$

where k is the extinction coefficient, and CHL_{M1}, CHL_{M2}, CHL_{BS}, and CHL_{M3} are chlorophyll contents of M1, M2, BS and M3 sections, respectively, and the size for each of these sections is calculated from structural parameters m and n_{BS} (Fig. 1). The total absorption by M cells ($I_{\mathrm{abs,M}}$) is the sum of $I_{\mathrm{abs,M1}}$, $I_{\mathrm{abs,M2}}$ and $I_{\mathrm{abs,M3}}$. Then, a_{M} and a_{BS} can be solved accordingly, and ($a_{\mathrm{M}}+a_{\mathrm{BS}}$) represents the whole-leaf absorptance, which can be used to fit the value of k. The $a_{\mathrm{BS}}/a_{\mathrm{M}}$ ratio, which is also the $I_{\mathrm{abs,BS}}/I_{\mathrm{abs,M}}$ ratio, can be expressed as:

$$\frac{a_{\mathrm{BS}}}{a_{\mathrm{M}}} = \frac{(1-m)e^{-k\cdot CHL_{\mathrm{M2}}}(1-e^{-k\cdot CHL_{\mathrm{BS}}})}{m(1-e^{-k\cdot CHL_{\mathrm{M1}}}) + (1-m)(1-e^{-k\cdot CHL_{\mathrm{M2}}}) + (1-m)e^{-k\cdot(CHL_{\mathrm{M2}}+CHL_{\mathrm{BS}})}(1-e^{-k\cdot CHL_{\mathrm{M3}}})} \quad \text{(C5)}$$

Table M2. Definition of symbols in the model

Symbol	Definition	Unit
a	Fraction of OAA that is reduced in M chloroplasts to malate	-
a_{BS}	Fraction of I_{inc} that is absorbed by BS chlorophyll pigments	-
a_{M}	Fraction of I_{inc} that is absorbed by M chlorophyll pigments	-
A_{g}	Gross rate of CO_2 assimilation	μmol CO_2 m^{-2} s^{-1}
$A_{\mathrm{g,atp}}$	ATP-determined gross rate of CO_2 assimilation	μmol CO_2 m^{-2} s^{-1}
$A_{\mathrm{g,nadph}}$	NADPH-determined gross rate of CO_2 assimilation	μmol CO_2 m^{-2} s^{-1}
[CHL]	Whole-leaf chlorophyll content	μmol CHL m^{-2}
C_{x}	Total amount of PSI used for CET	μmol PSI m^{-2}
$f_{\mathrm{atp,BS}}$	Fraction of ATP that is produced in BS cells	-
f_{bsCHL}	Fraction of [CHL] that is in BS cells	-
f_{bsPSI}	Fraction of PSI that is in BS cells	-
f_{CET}	Fraction of PSI electron flux being CET on the whole-leaf basis	-
$f_{\mathrm{CET,BS}}$	Fraction of PSI electron flux being CET on the BS-cell basis	-
$f_{\mathrm{CET,M}}$	Fraction of PSI electron flux being CET on the M-cell basis	-
f_{NDH}	Fraction of the whole-leaf CET that is NDH-dependent	-
$f_{\mathrm{nadph,BS}}$	Fraction of NADPH that is produced in BS cells	-
f_{Q}	Fraction of electrons at plastoquinone following the Q cycle	-
h	Number of protons (H^+) required per ATP synthesis	mol H^+ (mol ATP)$^{-1}$
H_{LET}	Number of protons (H^+) generated per electron along LET	mol H^+ (mol electron)$^{-1}$
H_{CET}	Number of protons (H^+) generated per electron along CET	mol H^+ (mol electron)$^{-1}$
I_{inc}	Incident irradiance	μmol photon m^{-2} s^{-1}

I_{abs}	Absorbed irradiance by whole-leaf chlorophyll pigments	μmol photon m^{-2} s^{-1}
$I_{abs,BS}$	Absorbed irradiance by BS-chlorophyll pigments	μmol photon m^{-2} s^{-1}
$I_{abs,M}$	Absorbed irradiance by M-chlorophyll pigments	μmol photon m^{-2} s^{-1}
J_{atp}	Whole-leaf ATP production rate	μmol ATP m^{-2} s^{-1}
$J_{atp,BS}$	ATP production rate in the BS cells	μmol ATP m^{-2} s^{-1}
$J_{CET,BS}$	Rate of cyclic electron transport rate around PSI in BS cells	μmol electron m^{-2} s^{-1}
$J_{CET,M}$	Rate of cyclic electron transport rate around PSI in M cells	μmol electron m^{-2} s^{-1}
$J_{LET,BS}$	Rate of linear electron transport in BS cells	μmol electron m^{-2} s^{-1}
$J_{LET,M}$	Rate of linear electron transport in M cells	μmol electron m^{-2} s^{-1}
J_{nadph}	Whole-leaf NADPH production rate	μmol NADPH m^{-2} s^{-1}
$J_{nadph,BS}$	NADPH production rate in the BS cells	μmol NADPH m^{-2} s^{-1}
k	Light extinction coefficient	m^2 (μmol CHL)$^{-1}$
k_{BS}	Photons absorbed per unit photosystem in BS cells	mol photon (mol PS)$^{-1}$
k_M	Photons absorbed per unit photosystem in M cells	mol photon (mol PS)$^{-1}$
m	Fraction of one unit interveinal distance for the M1 section in Fig. 1	-
n	ATP produced per NADH oxidation in mitochondria	mol ATP (mol NADH)$^{-1}$
n_{BS}	Fraction of one unit depth for the BS section in Fig. 1	-
p	Required ATP that is from LET	mol ATP (mol CO_2)$^{-1}$
T	Total amounts of PSII in BS and M cells	μmol PSII m^{-2}
u	Fraction of M cells-absorbed irradiance that is used for LET	-
v	Fraction of BS cells-absorbed irradiance that is used for LET	-
w	Required ATP for C_4 cycle that is from cyclic electron transport	mol ATP (mol CO_2)$^{-1}$
α	Fraction of PSII that is in BS cells	-
β	Fraction of used-for-CET PSI that is in BS cells	-
γ	Fraction of NADPH or ATP for 3-PGA reduction in BS cells	-
ϕ	Leakiness	-
Φ_{1LL}	Quantum yield of PSI photochemistry under limiting light	mol electron (mol photon)$^{-1}$
Φ_{2LL}	Quantum yield of PSII photochemistry under limiting light	mol electron (mol photon)$^{-1}$
Φ_{CO2}	Quantum yield of whole-leaf CO_2 assimilation	mol CO_2 (mol photon)$^{-1}$
η	Fraction of OAA that follows the primary decarboxylation pathway	-
η_{CET}	Efficiency of CET in converting absorbed light into ATP	mol ATP (mol photon)$^{-1}$
η_{LET}	Efficiency of LET in converting absorbed light into ATP	mol ATP (mol photon)$^{-1}$
φ	Extra chloroplastic ATP required per C_4 cycle	mol ATP (mol CO_2)$^{-1}$
$\upsilon_{n/c}$	Ratio of nitrate reduction to RuBP carboxylation	-
$\upsilon_{o/c}$	Ratio of RuBP oxygenation to RuBP carboxylation	-
$\upsilon_{r/c}$	Ratio of day respiration to RuBP carboxylation	-

Methods S2 FST codes of our model for NADPH and ATP production and quantum yield

The source codes listed below are written using FST (FORTRAN Simulation Translator), software that is freely downloadable at the site (http://models.pps.wur.nl/node/970). The FST is a powerful and easy-to-use simulation language providing clear error message (van Kraalingen et al. 2003). Output variables under its PRINT statement can be shown in both tabular and chart forms. The codes provided herein can be copied and pasted to the FST editor window, where the codes need to be saved as an .FST file before it can be run. Alternatively, an electronic copy of the FST codes can be obtained upon request from the corresponding author. The codes can also be converted if readers are used to other modelling software platforms. Two intrinsic FST functions, INSW and FCNSW, used in the codes need then to be re-written. The mathematical meaning of Y = INSW(X, Y1, Y2) is: Y = Y1 if X < 0, and Y = Y2 if X ≥ 0. The mathematical meaning of Y = FCNSW(X, Y1, Y2, Y3) is: Y = Y1 if X < 0, Y = Y2 if X = 0, and Y = Y3 if X > 0. An alphabetical list of variables used in the codes and their meaning is given after the codes.

```
****************************************************************************
*  A model for cell-type specific electron transport of C4 photosynthesis  *
*                                                                          *
*  Physiological principles of this model are explained in Methods S1 of  *
*  Supporting Information accompanying the paper by Yin & Struik (2018),   *
*  entitled "The energy budget in C4 photosynthesis: Insights from a       *
*  cell-type specific electron transport model". New Phytologist           *
*                                                                          *
*  Please note that while variables for fluxes (e.g., JLET, JATP, Ag,...)  *
*  are given in Methods S1 for the convenience of explanation, they are    *
*  not calculated in the codes, where physiological variables such as      *
*  quantum yield/efficiency, ratios, fractions, and etc, are modelled. In  *
*  this way, there is no need to use incident irradiance (Iinc) as input.  *
****************************************************************************

TITLE CELL TYPE-SPECIFIC C4 PHOTOSYNTHETIC ELECTRON TRANSPORT

*---Specifying a C4 type
*   PEPCK =1. for the standard PEP-CK subtype; =-1. for other types
*   PUREPEPCK =1. for the 'pure PEP-CK' type; =0. for the mixed ME + PEPCK type;
*             =-1. for NAD(P)-ME subtypes and the mixed NADP-ME + aspartate-malate type.
PARAM PEPCK = -1.
PARAM PUREPEPCK = -1.

*---Input parameters
PARAM H=4.; FQ=1.; FNDH=0.
PARAM PHI2LL =0.8; PHI21=0.85
PARAM NADPHreqb=2.; phi = 0.16
PARAM K = 0.005
PARAM M=0.55; Nbs=0.6

PARAM CHLleaf=579.; FbsCHL=0.33
PARAM FbsPSI = 0.37
PARAM ALPHA  = 0.01
*Note that ALPHA cannot equal FbsPSI (explained in the text after eqn B10 of Methods S1)

*---Relative area of four section components in Figure 1
      Nmad    = (1.-Nbs)/2.
```

```
      Nmab    = 1.-Nmad-Nbs
      AREAm   = M + (1.-M)*Nmad + (1.-M)*Nmab
      AREAbs  = (1.-M)*Nbs
      AreaBST = AREABS/(AREAm+AREAbs)

*---Chlorophyll content of each section component
      CHLbs   = CHLleaf*    FbsCHL
      CHLm    = CHLleaf*(1.-FbsCHL)
      CHL1M   =     M                /AREAm * CHLm
      CHL2MAD = (1.-M)*Nmad          /AREAm * CHLm
      CHL2MAB = (1.-M)*(1.-Nmad-Nbs)/AREAm * CHLm

*---Light absorption of each component
      ABS1M   =     M *(1.-exp(-K*CHL1M))
      ABS2MAD = (1.-M)*(1.-exp(-K*CHL2MAD))
      ABS2MAB = (1.-M)*(1.-exp(-K*CHL2MAB))*EXP(-K*(CHL2MAD+CHLbs))

      ABSmc   = ABS1M + ABS2MAD + ABS2MAB
      ABSbs   = (1.-M)*(1.-exp(-K*CHLbs))*EXP(-K*CHL2MAD)
      ABSTOT  = ABSmc + ABSbs

*---Ratio of BS:M absorption
      Absm    = ABSbs/ABSmc

*---Protons (H+) produced per electron transferred by LET and CET
      HLET    = 2.+FQ
      HCET    = 1.+FQ + 2.*FNDH

*---Required ATP that is produced from LET (PQ) and that is from CET (W)
PARAM n = 3.
      VOC  = 1./20.
      VNC  = 1./35.
      RDC  = 1./40.
      STARCH = 2./12.
      aaa    = 1./(1.+n)
      eta    = 0.7

      ATPPEP  = FCNSW(PUREPEPCK, 2., 1.+eta, 1.)
      j       = INSW(PEPCK, ATPPEP, 2.*aaa)
      NADPHreq= NADPHreqb + INSW(PEPCK, 0., aaa*(1.+phi))
      IV      = -0.5*VOC +10.*VNC*(2.+FQ)/H-1.*VNC -STARCH*(1.-0.5*VOC-RDC)
      atpadd  = INSW(PEPCK, 0., (2.+FQ)/H*(2.*aaa*(1.+phi))) + IV

      W       = j*(1.+phi)+(3.-4.*(2.+FQ)/H)*(1.+VOC) - atpadd
      PQ      = (2.*(NADPHreq+2.*VOC)+10.*VNC)*(2.+FQ)/H

*---Some intermediate variables
      B       = Absm*((1.-ALPHA)*(1.+PHI21) - PHI21*(FbsPSI-ALPHA))
      C       = Absm*((FbsPSI-ALPHA)*PHI21 - (1.-ALPHA)*FbsPSI*(1.+PHI21))
      D       = ALPHA  + PHI21*FbsPSI
      E       = -ALPHA*FbsPSI*(1.+PHI21)

      F       = -((FbsPSI-ALPHA)*HCET*PQ/(HLET*W)+ (1.-ALPHA))
      G       =   (FbsPSI-ALPHA)*HCET*PQ/(HLET*W)+ (1.-ALPHA)*FbsPSI
      II      = ALPHA - (FbsPSI-ALPHA)*HCET*PQ/(HLET*W)
      JJ      = -ALPHA*FbsPSI

      AA      = B*II - D*F
      BB      = B*JJ + C*II - D*G - E*F
      CC      = C*JJ - E*G

*---Solution to Beta
      BETA1   = (-BB+(MAX(0.,BB**2-4.*AA*CC))**0.5)/(2.*AA)
      BETA2   = (-BB-(MAX(0.,BB**2-4.*AA*CC))**0.5)/(2.*AA)
      BETA    = INSW (ALPHA-FbsPSI, BETA1, BETA2)

*---Kbs : Km ratio
      KbsKm   = (F*BETA+G)/(II*BETA+JJ)

*---PSI(used for CET) : PSII ratio
      CxT    = (HLET*W)/HCET/PQ*PHI21*(Kbskm*ALPHA+(1.-ALPHA))/(Kbskm*BETA+(1.-BETA))
```

```
*---PS(BS) : PS(M) ratio
      PSbsm   = Absm/KbsKm

*---PSI : PSII ratio
      PS12    = PHI21 + CxT
      PS12BS  = PHI21 +     BETA /    ALPHA *CxT
      PS12M   = PHI21 + (1.-BETA)/(1.-ALPHA)*CxT

*---Fraction of BS-absorbed light used for LET (V) and that of M-absorbed light for LET (U)
      IM      = (HLET*W)/(HCET*PQ)*(KbsKm*ALPHA+(1.-ALPHA))/(KbsKm*BETA+(1.-BETA))
      V       = (1.+PHI21)/(1.+PHI21*(1.+IM*    BETA /    ALPHA))
      U       = (1.+PHI21)/(1.+PHI21*(1.+IM*(1.-BETA)/(1.-ALPHA)))

*---Quantum efficiency of electron transport rates on the whole-leaf incident light basis
      QLETBS  = PHI2LL/(1.+PHI21)*V*ABSbs
      QLETM   = PHI2LL/(1.+PHI21)*U*ABSmc
      QCETBS  = PHI2LL/PHI21*(1.-V)*ABSbs
      QCETM   = PHI2LL/PHI21*(1.-U)*ABSmc

      QBS     = QLETBS+QCETBS
      QM      = QLETM +QCETM

*---BS:M ratio in total electron transport
      ReBSM   = QBS/QM

*---Fraction of CET (whole-leaf, BS, M, respectively)
      FCET    = (QCETBS+QCETM)/(QLETBS+QLETM+QCETBS+QCETM)
      FCETBS  =  QCETBS/QBS
      FCETM   =  QCETM /QM

*---Ratio of CET in BS : CET in total
      RcetBS  =  QCETBS /(QCETBS+QCETM)

*---Ratio of BS ATP from CET : total BS ATP
      RaCBS    =  HCET/H*QCETBS / (HLET/H*QLETBS+HCET/H*QCETBS)

*---Quantum efficiency of NADPH and ATP production rates on the whole-leaf incident light basis
      QJNADPH = 0.5*(QLETBS+QLETM)
      QJATP   = HLET/H*(QLETBS+QLETM) + HCET/H*(QCETBS+QCETM)

*---Ratio of ATP produced by LET to the total ATP
      RATPLET = HLET/H*(QLETBS+QLETM)/QJATP

*---Ratio of ATP or NADPH produced in BS to that in the whole-leaf
      RATPBS  = (HLET/H*QLETBS+HCET/H*QCETBS)/QJATP
      RNADPHBS= 0.5*QLETBS/QJNADPH

*---Quantum yield of CO2 assimilation on the whole-leaf absorbed light basis
      FCO2n  = QJNADPH/(NADPHreq + 2.*VOC + 5.*VNC) /ABSTOT
      FCO2a  = QJATP  /(3. + 3.5*VOC + j*(1.+phi) +1.*VNC +STARCH*(1.-0.5*VOC-RDC)) /ABSTOT
      FCO2   = MIN(FCO2n,FCO2a)

*---Intrinsic FST run-time control
TIMER STTIME=1.; FINTIM=50.; DELT=1.; PRDEL=1.

*---Printing the output of calculated variables
PRINT AreaBST,ABSTOT,Absm,ALPHA,BETA1,BETA2,BETA,U,V,CxT,KbsKm,PSbsm,PS12,PS12BS,PS12M,...
      ReBSM,FCET,FCETBS,FCETM,RcetBS,RNADPHBS,RATPBS,RaCBS,RATPLET,FCO2n,FCO2a,FCO2
END

*---Reruns with respect to input parameters (here: to represent different C4 species)
PARAM CHLleaf=464.; FbsCHL=0.38
PARAM FbsPSI = 0.39
PARAM ALPHA  = 0.04
END
PARAM CHLleaf=424.; FbsCHL=0.6
PARAM FbsPSI = 0.24
PARAM ALPHA  = 0.17
END
PARAM CHLleaf=425.; FbsCHL=0.59
```

```
PARAM FbsPSI = 0.46
PARAM ALPHA  = 0.35
END
STOP
```

A full list of variables used in the above source codes, and their corresponding model symbols, definitions and units

Variable	Symbol	Definition	Unit
AA	A	An intermediate variable	-
aaa	a	Extra NADPH required for CCM in the standard PEP-CK subtype	mol NADPH (mol CO_2)$^{-1}$
Absm	a_{BS}/a_M	BS:M ratio in light absorption	-
ABS1M		Light absorptance by the M1 section in Fig. 1	-
ABS2MAB		Light absorptance by the M3 section in Fig. 1	-
ABS2MAD		Light absorptance by the M2 section in Fig. 1	-
ABSbs		Light absorptance by the BS section in Fig. 1	-
ABSmc		Light absorptance by the M sections in Fig. 1	-
ABSTOT		Light absorptance by both BS and M sections	-
ALPHA	α	Fraction of total PSII that is in BS cells	-
AREAbs		Relative area of the BS section in Fig. 1	-
AreaBST		Area ratio of the BS section to the total in Fig. 1	-
AREAm		Relative area of the M sections in Fig. 1	-
atpadd		ATP produced from additional LET due to extra NADPH required in the standard PEP-CK subtype and due to alternative e^- sinks	mol ATP (mol CO_2)$^{-1}$
ATPPEP		Chloroplastic ATP required for PEP regeneration in C_4 cycle (for types other than the standard PEP-CK subtype)	mol ATP (mol CO_2)$^{-1}$
B	b	An intermediate variable	-
BB	B	An intermediate variable	-
BETA	β	Fraction of used-for-CET PSI that is in BS cells	-
BETA1		First solution to BETA	-
BETA2		Second solution to BETA	-
C	c	An intermediate variable	-
CC	C	An intermediate variable	-
CHL1M		Chlorophyll content in the M1 section in Fig. 1	μmol CHL m^{-2}
CHL2MAB		Chlorophyll content in the M3 section in Fig. 1	μmol CHL m^{-2}
CHL2MAD		Chlorophyll content in the M2 section in Fig. 1	μmol CHL m^{-2}
CHLbs		Chlorophyll content in the BS section in Fig. 1	μmol CHL m^{-2}
CHLleaf	[CHL]	Leaf chlorophyll content	μmol CHL m^{-2}
CHLm		Chlorophyll content in the M sections in Fig. 1	μmol CHL m^{-2}
CxT	C_x/T	Ratio of PSI used for CET to total PSII	-
D	d	An intermediate variable	-
E	e	An intermediate variable	-
eta	η	Fraction of OAA that follows the primary decarboxylation pathway	-
F	f	An intermediate variable	-
FbsCHL	f_{bsCHL}	Fraction of leaf chlorophyll that is in BS cells	-
FbsPSI	f_{bsPSI}	Fraction of total PSI that is in BS cells	-
FCET	f_{CET}	Fraction of total electron transport that is CET	-
FCETBS	$f_{CET,BS}$	f_{CET} in BS cells	-
FCETM	$f_{CET,M}$	f_{CET} in M cells	-
FCO2	Φ_{CO2}	Quantum yield of CO_2 assimilation on the absorbed light basis	mol CO_2 (mol photon)$^{-1}$
FCO2a		ATP production determined Φ_{CO2}	mol CO_2 (mol photon)$^{-1}$
FCO2n		NADPH production determined Φ_{CO2}	mol CO_2 (mol photon)$^{-1}$
FNDH	f_{NDH}	Fraction of total CET that follows the NDH-dependent pathway	-
FQ	f_Q	Fraction of electrons at plastoquinone that follow the Q cycle	-
G	g	An intermediate variable	-
H	h	Number of protons (H^+) required per ATP synthesised	mol H^+ (mol ATP)$^{-1}$
HCET	H_{CET}	Number of protons (H^+) produced per electron transferred by CET	mol H^+ (mol e^-)$^{-1}$
HLET	H_{LET}	Number of protons (H^+) produced per electron transferred by LET	mol H^+ (mol e^-)$^{-1}$

II	*i*	An intermediate variable	-
IM		An intermediate variable	-
IV		An intermediate variable	mol ATP (mol CO_2)$^{-1}$
j	φ	Chloroplastic ATP required per C_4 cycle	mol ATP (mol CO_2)$^{-1}$
JJ	*j*	An intermediate variable	-
K	*k*	Light extinction coefficient	m^2 (μmol CHL)$^{-1}$
KbsKm	k_{BS}/k_M	BS:M absorptance ratio per photosystem	-
M	*m*	Fraction of a unit interveinal distance for the M1 section in Fig. 1	-
n	*n*	ATP produced per NADH oxidised in mitochondrial electron transport chain	mol ATP (mol NADH)$^{-1}$
NADPHREQ		NADPH required per CO_2 assimilated	mol NADPH (mol CO_2)$^{-1}$
NADPHREQb		NADPH required per CO_2 assimilated in types other than the standard PEP-CK subtype (= 2)	mol NADPH (mol CO_2)$^{-1}$
Nbs	n_{BS}	Fraction of one unit depth for the BS section in Fig. 1	-
Nmab	n_{Mab}	Fraction of one unit depth for the M3 section in Fig. 1	-
Nmad	n_{Mad}	Fraction of one unit depth for the M2 section in Fig. 1	-
PEPCK		A code to indicate C_4 types: 1. to represent the standard PEP-CK subtype, and -1. to represent any other types	
phi	ϕ	Leakiness	-
PHI2LL	Φ_{2LL}	Photochemical efficiency of PSII under limiting light	mol e^- (mol photon)$^{-1}$
PHI21	Φ_{2LL}/Φ_{1LL}	Ratio of PSII : PSI photochemical efficiency under limiting light	-
PQ	*p*	Required ATP that is from LET	mol ATP (mol CO_2)$^{-1}$
PS12	*PSI:PSII*	Total PSI to total PSII ratio	-
PS12BS		PSI:PSII ratio in BS cells	-
PS12M		PSI:PSII ratio in BS cells	-
PSbsm		BS:M ratio in total photosystems	-
PUREPEPCK		A code to indicate C_4 types: 1. to represent the pure PEP-CK type, 0. to represent the mixed "ME + PEP-CK" types, and -1. to represent any other types	
QBS		Quantum efficiency of electron transport in BS cells on incident light basis	mol e^- (mol photon)$^{-1}$
QCETBS		Quantum efficiency of CET in BS cells on incident light basis	mol e^- (mol photon)$^{-1}$
QCETM		Quantum efficiency of CET in M cells on incident light basis	mol e^- (mol photon)$^{-1}$
QLETBS		Quantum efficiency of LET in BS cells on incident light basis	mol e^- (mol photon)$^{-1}$
QLETM		Quantum efficiency of LET in M cells on incident light basis	mol e^- (mol photon)$^{-1}$
QJNADPH		Quantum efficiency of NADPH production on incident light basis	mol NADPH (mol photon)$^{-1}$
QJATP		Quantum efficiency of ATP production on incident light basis	mol ATP (mol photon)$^{-1}$
QM		Quantum efficiency of electron transport in M cells on incident light basis	mol e^- (mol photon)$^{-1}$
RaCBS		Ratio of BS ATP from CET to total BS ATP	-
RATPBS		Ratio of ATP in BS cells to total ATP	-
RATPLET		Ratio of ATP from LET to the total ATP	-
RcetBS		Ratio of CET in BS cells to CET total	-
RDC	$\upsilon_{r/c}$	Day-respiration to carboxylation ratio	-
ReBSM		BS:M ratio in total electron transport	-
RNADPHBS		Ratio of NADPH in BS cells to total NADPH	-
STARCH	c_{starch}	ATP cost for starch synthesis	mol ATP (mol CO_2)$^{-1}$
U	*u*	Fraction of M cells-absorbed irradiance that is used for LET	-
V	*v*	Fraction of BS cells-absorbed irradiance that is used for LET	-
VNC	$\upsilon_{n/c}$	Nitrate-reduction to carboxylation ratio	-
VOC	$\upsilon_{o/c}$	Oxygenation to carboxylation ratio	-
W	*w*	Required ATP for C_4 cycle that is from CET	mol ATP (mol CO_2)$^{-1}$

Impact of uncertainties in some input parameters on model results

The default values of structure parameters in Table 1 (m = 0.55, n_{BS} = 0.6) were determined based on the literature (Christin et al. 2013; Griffiths et al. 2013; Bellasio & Lundgren 2016) as an average of C_4 species. According to our simple scheme in Fig. 1, these default values yield a BS:(BS+M) area ratio of 0.27 as an average of diverse C_4 species (Griffiths et al. 2013). However, significant variation of this ratio exists among species (Hattersley 1984), with the NAD-ME species having higher ratios than the NADP-ME species. For example, this ratio was 0.29 and 0.39 for two NAD-ME species *P. miliaceum* and *P. coloratum*, respectively, versus 0.21 and for 0.23 for *Zea mays* and *S. bicolor*, respectively (Hattersley 1984). This difference was largely due to the higher mesophyll area per vein (determined by parameter m in Fig. 1) in NAD-ME than in NADP-ME and PEP-CK subtypes (Hattersley 1984). Such a difference between subtypes may be counteracted, to some extent, by the fact that the arrangement of BS chloroplasts is centripetal in NAD-ME, and centrifugal in NADP-ME and PEP-CK species (von Caemmerer & Furbank 2003). This may support using a common default m:(1−m) as the interveinal mesophyll:vein surface area ratio (Fig. 1) across species for the modelling purpose. However, as some model output parameters are sensitive to parameter m, it is necessary to analyse the impact of potential uncertainties in m on model outputs. To this end, we run the model with up to ± 40% change of m or n_{BS} relative to their default values, with which an almost full range of the reported variation among C_4 species in the BS:(BS+M) area ratio (Hattersley 1984) could be reached. Similarly, the value of light extinction coefficient k (Table 1) also affects the relative absorptance between M and BS cells, and therefore, a sensitivity analysis with respect to k is necessary. All the modelled results of sensitivity analysis shown below are for the case in the presence of photorespiration and alternative energy-using sinks.

Notes S1 The effect of structure parameters on modelled fraction of CET in each cell type for the NAD-ME species

We have shown in the main text that using the default values of m or n_{BS}, the predicted PSI:PSII ratio and f_{CET} in BS cells were undoubtedly higher than in M cells in the two NADP-ME species;

but they were also higher in the two NAD-ME species (Table 2). This latter prediction differs from the statement of Takabayashi et al. (2005) that the activity of CET and the PSI:PSII ratio should be higher in M cells than in BS cells of NAD-ME species, opposite to that shown for the NADP-ME species. We examine to what extent our result varies with different sets of structural parameter values (Table S1).

The modelled values of both PSI:PSII ratio and f_{CET} were still higher in BS than in M cells of the two NAD-ME species (Table S1), irrespective of the changes in either m or n_{BS} for a higher BS:(BS+M) area ratio that is often observed for NAD-ME species. So, our conclusion with regard to differences from Takabayashi et al. (2005) still holds.

Table S1 The modelled PSI:PSII ratio and fraction of total electron flux that is CET (f_{CET}) in each cell type of the two NAD-ME species in response to a ±40% change of input parameters m or n_{BS}. Values for the default case are also shown in Table 2.

		BS:(BS+M) area ratio	*Panicum miliaceum*				*Panicum coloratum*			
			PSI:PSII		f_{CET}		PSI:PSII		f_{CET}	
			BS	M	BS	M	BS	M	BS	M
Default m & n_{BS}		0.270	2.18	1.41	0.61	0.40	2.21	1.40	0.62	0.39
m	+40%	0.138	2.38	1.54	0.64	0.45	2.35	1.55	0.64	0.45
	−40%	0.402	2.07	1.34	0.59	0.37	2.06	1.36	0.59	0.37
n_{BS}	+40%	0.378	2.17	1.41	0.61	0.40	2.15	1.42	0.61	0.40
	−40%	0.162	2.18	1.42	0.61	0.40	2.17	1.43	0.61	0.40

Notes S2 The effect of structure parameters on the estimated requirement of the "aspartate-malate" mechanism as the secondary decarboxylating pathway in NADP-ME species

We have shown in the main text the estimated η (the required fraction of C_4 acids that follow the primary decarboxylating pathway) if the "aspartate-malate" mechanism acts as the secondary decarboxylating route in the two NADP-ME species. The solved value of η was 0.68-0.73 (Table 5), when using the default values of input parameters m and n_{BS}. In a sensitivity analysis here, we initially tried to change either input parameter by ± 40% to check if the estimated η still holds. It turned out that parameter m cannot be decreased by more than 18%, especially for *Cenchrus*

ciliaris, as the model predicted *u* (the fraction of absorbed light by M cells that is used to drive LET) and solved η would otherwise have been > 1.0. This indicates that our model can be used to define the limits of variation in structural parameters conditional to a given set of physiological input parameters [CHL], f_{bsCHL}, α and f_{bsPSI}. The analysis also showed that the model output is less sensitive to a change in n_{BS}. Thus, for the result of sensitivity analysis shown here, we set changes of ± 18% for *m*, while changes in n_{BS} remained ± 40% (Table S2).

The calculated $f_{nadph,BS}$ varied little, but $f_{atp,BS}$ did vary significantly, with a change in structural parameters. As a consequence, the solved γ for ATP and η varied as well (Table S2). However, solved γ for ATP was always lower than solved γ for NADPH (except one extreme case where they were nearly equal for *Cenchrus ciliaris* with the BS:(BS+M) area ratio set high for the NADP-ME type), suggesting that a secondary decarboxylating pathway is generally required for NADP-ME species. But the quantitative extent for this secondary pathway had a wide range of variation, depending more on *m* than on n_{BS}, even wider than what was discussed in the main text for reported ranges of variation in the fraction for the initial carbon label partitioned to aspartate ($1-\eta$), i.e. from ca 25% in maize (Hatch 1971) to ca 50% for *Flaveria bidentis* (Meister et al. 1996).

Therefore, whether the "aspartate-malate" mechanism as the secondary decarboxylating pathway is required, and if so, to what extent it operates, may depend on species within the NADP-ME subtype.

Table S2 The modelled fraction of total NADPH or ATP that is produced in BS cells ($f_{nadph,BS}$ or $f_{atp,BS}$), calculated γ (the fraction of required NADPH or ATP for the reduction phase of the Calvin circle that is consumed in BS cells) and the required fraction (η) of C_4 acids that follow the primary decarboxylation if the "aspartate-malate" mechanism acts as the secondary decarboxylating pathway in two NADP-ME species, in response to a certain % change of parameters *m* or n_{BS}. Values for the default case are also shown in Table 5.

		BS:(BS+M) area ratio	*Sorghum bicolor*					*Cenchrus ciliaris*				
			$f_{nadph,BS}$	$f_{atp,BS}$	γ		η	$f_{nadph,BS}$	$f_{atp,BS}$	γ		η
					NADPH	ATP				NADPH	ATP	
Default *m* & n_{BS}		0.270	0.01	0.36	0.56	0.38	0.68	0.06	0.39	0.61	0.46	0.73
m	+18%	0.210	0.01	0.29	0.56	0.20	0.37	0.04	0.32	0.59	0.28	0.44
	−18%	0.330	0.02	0.40	0.57	0.50	0.88	0.07	0.45	0.62	0.63	1.00
n_{BS}	+40%	0.378	0.02	0.43	0.57	0.51	0.90	0.07	0.43	0.62	0.57	0.92
	−40%	0.162	0.02	0.33	0.56	0.30	0.50	0.05	0.37	0.60	0.42	0.67

Notes S3 The effect of structure parameters on the estimated requirement of the "PEP-CK" mechanism as the secondary decarboxylating pathway in NADP-ME and NAD-ME species

We have shown in the main text that the PEP-CK mechanism alone acts as the secondary decarboxylation route, hardly for the NADP-ME subtype but well for the NAD-ME subtype. We now examine whether this conclusion can be affected by the uncertainties in input structural parameters m and n_{BS}. Again, we used the criteria that the predicted u or v must be within the physiologically relevant range ($0 \leq u \leq 1$ and $0 \leq v \leq 1$) to define the range of variation in m and n_{BS}. It turned out that parameter m cannot be decreased by more than 2.5% and n_{BS} cannot be increased by more than 13% in the NADP-ME species when involving the PEP-CK as the secondary decarboxylation, as the modelled u would otherwise be > 1. This little allowed decrease in m reflects the stiff system once involving PEP-CK, echoing what has been shown in the main text that the allowable range of parameter α in the PEP-CK subtype is very narrow (Table 3). However, the NAD-ME species were not very sensitive as shown in Table S1 where m could be allowed to vary by ± 40% and beyond. So, our model predicts a higher phenotypic plasticity of structural parameters in NAD-ME than NADP-ME subtypes. Given this high sensitivity in the NADP-ME species, we varied n_{BS} by ± 13% and set the lower limit of m as −2.5% while leaving its upper limit still as +18% of its default as in Table S2. The results are given in the upper and lower parts of Table S3 for the NADP-ME and NAD-ME species, respectively.

Using the changes made for parameter m or n_{BS}, the solved η in the NADP-ME species was always above 1 (the upper part of Table S3), which is physiologically impossible as discussed in the main text. This confirms that PEP-CK alone cannot act as the secondary decarboxylation pathway; it either does not exist, or co-acts with the "aspartate-malate" mechanism, in these species.

The solved η values, using the changes made for parameter m or n_{BS}, were all physiologically sensible for the NAD-ME species (the lower part of Table S3). But η depends on the structural parameters: more on m than on n_{BS}. When m was increased by 18%, the obtained η was 0.93-0.95, suggesting the required PEP-CK as secondary decarboxylation pathway was quite small. However, this decreased m corresponds to the BS:(BS+M) area ratio of 0.21 (Table S3), a low value hardly found for an NAD-ME species (Hattersley 1984).

Table S3 The modelled fraction of total NADPH or ATP that is produced in BS cells ($f_{nadph,BS}$ or $f_{atp,BS}$), calculated γ (the fraction of required NADPH or ATP for the reduction phase of the Calvin circle that is consumed in BS cells) and the required fraction (η) of C_4 acids that follow the primary decarboxylation if the "PEP-CK" mechanism acts as the secondary decarboxylating pathway in two NADP-ME species (the upper part of this table) and in two NAD-ME species (the lower part of the table), in response to a certain percent change of input parameters *m* or n_{BS}. Values for the default case are also shown in Table 5.

		BS:(BS+M) area ratio	*Sorghum bicolor*					*Cenchrus ciliaris*				
			$f_{nadph,BS}$	$f_{atp,BS}$	γ		η	$f_{nadph,BS}$	$f_{atp,BS}$	γ		η
					NADPH	ATP				NADPH	ATP	
Default *m* & n_{BS}		0.270	0.01	0.36	0.56	0.38	1.88	0.06	0.39	0.61	0.46	1.56
m	+18%	0.210	0.01	0.30	0.56	0.21	3.08	0.04	0.32	0.59	0.29	2.7
	−2.5%	0.278	0.01	0.37	0.56	0.41	1.76	0.06	0.41	0.61	0.51	1.46
n_{BS}	+13%	0.306	0.02	0.38	0.56	0.42	1.67	0.06	0.41	0.61	0.51	1.45
	−13%	0.234	0.01	0.35	0.56	0.36	2.06	0.06	0.39	0.61	0.47	1.66
		BS:(BS+M) area ratio	*Panicum miliaceum*					*Panicum coloratum*				
			$f_{nadph,BS}$	$f_{atp,BS}$	γ		η	$f_{nadph,BS}$	$f_{atp,BS}$	γ		η
					NADPH	ATP				NADPH	ATP	
Default *m* & n_{BS}		0.270	0.41	0.50	0.43	0.76	0.75	0.40	0.49	0.42	0.74	0.78
m	+18%	0.210	0.33	0.41	0.35	0.53	0.93	0.32	0.41	0.34	0.51	0.95
	−2.5%	0.278	0.42	0.51	0.45	0.79	0.73	0.41	0.50	0.44	0.77	0.76
n_{BS}	+13%	0.306	0.42	0.50	0.44	0.77	0.75	0.41	0.50	0.43	0.75	0.77
	−13%	0.234	0.41	0.50	0.43	0.76	0.76	0.40	0.49	0.42	0.74	0.78

Notes S4 The effect of light extinction coefficient *k* on the estimated requirement of the secondary decarboxylating pathway in NADP-ME and NAD-ME species

The default *k* value of 0.005 m^2 (μmol CHL)$^{-1}$ (Table 1) was obtained from fitting the light absorptance model, eqns (C1-C4), to have a good modelled whole-leaf absorptance. The value of *k* has an effect not only on the whole-leaf absorptance but also on the relative absoprtance of light between M and BS cells, thereby, affecting the fraction of NADPH or ATP that is produced in BS cells. Also, Bellasio & Griffiths (2014) showed that "blue light was strongly absorbed (steep profile), green light was weakly absorbed (gradual profile), while red light had an intermediately profile of light penetration", indicating that value of *k* depends on the light spectrum. Given these uncertainties of the *k* value, we conducted a sensitivity analysis by varying *k* by ± 40% of its default value, to check to what extent the key result of this paper on the

Table S4 The modelled fraction of total NADPH or ATP that is produced in BS cells ($f_{nadph,BS}$ or $f_{atp,BS}$), calculated γ (the fraction of the required NADPH or ATP for the reduction phase of the Calvin circle that is consumed in BS cells) and the required fraction (η) of C_4 acids that follow the primary decarboxylation, when the secondary decarboxylating pathway was the "aspartate-malate" mechanism in two NADP-ME species (the upper part of the Table) and the "PEP-CK" mechanism in two NAD-ME species (the lower part of the Table), in response to a certain percent change in the value of the input parameter light extinction coefficient *k*. Values for the default case are also shown in Table 5.

		Sorghum bicolor					*Cenchrus ciliaris*				
		$f_{nadph,BS}$	$f_{atp,BS}$	γ		η	$f_{nadph,BS}$	$f_{atp,BS}$	γ		η
				NADPH	ATP				NADPH	ATP	
Default *k*=0.005		0.01	0.36	0.56	0.38	0.68	0.06	0.39	0.61	0.46	0.73
k	+40%	0.01	0.34	0.56	0.33	0.59	0.05	0.38	0.60	0.43	0.70
	−40%	0.01	0.37	0.56	0.40	0.71	0.06	0.41	0.61	0.51	0.81
		Panicum miliaceum					*Panicum coloratum*				
		$f_{nadph,BS}$	$f_{atp,BS}$	γ		η	$f_{nadph,BS}$	$f_{atp,BS}$	γ		η
				NADPH	ATP				NADPH	ATP	
Default *k*=0.005		0.41	0.50	0.43	0.76	0.75	0.40	0.49	0.42	0.74	0.78
k	+40%	0.38	0.47	0.40	0.68	0.82	0.37	0.46	0.39	0.66	0.84
	−40%	0.45	0.53	0.47	0.85	0.69	0.43	0.52	0.46	0.83	0.71

distribution of 3-PGA distribution and the required fraction of the secondary decarboxylating pathway would vary in NADP-ME and NAD-ME species.

As expected, with a change in *k*, the modelled whole-leaf absorptance varied significantly, but the relative absorptance between M and BS cells varied less (results not shown) because the latter depended more on the relative M:BS distribution of chlorophyll. As a result, the modelled fraction of total NADPH or ATP that is produced in BS cells ($f_{nadph,BS}$ or $f_{atp,BS}$), the calculated γ (the fraction of required NADPH or ATP for the reduction phase of the Calvin circle that is consumed in BS cells) and the required fraction (η) of C_4 acids that follow the primary decarboxylation, all only varied marginally in response to *k* (Table S4). Similar marginal impacts were modelled for some intermediate variables (results not shown). Therefore, our key quantitative estimates in the main text on the distribution of the 3-PGA reduction and the requirement of the secondary decarboxylation are conservative to an uncertainty in light extinction coefficient *k*. Needless to say, *k* has no impact on the calculation of the whole-leaf f_{CET} and Φ_{CO2}.

References

Amthor JS. 2010. From sunlight to phytomass: on the potential efficiency of converting solar radiation to phyto-energy. *New Phytologist* **188:** 939-959.

Bellasio C. 2017. A generalized stoichiometric model of C_3, C_2, C_2+C_4, and C_4 photosynthetic metabolism. *Journal of Experimental Botany* **68:** 269-282.

Bellasio C, Griffiths H. 2014. The operation of two decarboxylase, transamination, and partitioning of C_4 metabolic processes between mesophyll and bundle sheath cells allows light capture to be balanced for the maize C_4 pathway. *Plant Physiology* **164:** 466-480.

Bellasio C, Lundgren MR. 2016. Anatomical constraints to C_4 evolution: light harvesting capacity in the bundle sheath. *New Phytologist* **212:** 485-496.

Christin P-A, Osborne CP, Chatelet DS, Columbus T, Besnard G, Hodkinson TR, Garrison LM, Vorontsova MS, Edwards EJ. 2013. Anatomical enablers and the evolution of C_4 photosynthesis in grasses. *Proceedings of the National Academy of Sciences of USA* **110:** 1381-1386.

Evans JR. 1995. Carbon fixation profiles do reflect light absorption profiles in leaves. *Australian Journal of Plant Physiology* **22:** 865-873.

Farquhar GD. 1983. On the nature of carbon isotope discrimination in C_4 species. *Australian Journal of Plant Physiology* **10:** 205-226.

Farquhar GD, von Caemmerer S, Berry JA. 1980. A biochemical model of photosynthetic CO_2 assimilation in leaves of C_3 species. *Planta* **149:** 78-90.

Furbank RT, Jenkins CLD, Hatch MD. 1990. C_4 photosynthesis: Quantum requirement, C_4 acid overcycling and Q-cycle involvement. *Australian Journal of Plant Physiology* **17:** 1-7.

Ghannoum O, Evans JR, Chow WS, Andrews J, Conroy J, von Caemmerer S. 2005. Faster Rubisco is the key to superior nitrogen-use efficiency in NADP-malic enzyme relative to NAD-malic enzyme C_4 grasses. *Plant Physiology* **137:** 638-650.

Griffiths H, Weller G, Toy LFM, Dennis RJ. 2013. You're so vein: bundle sheath physiology, phylogeny and evolution in C_3 and C_4 plants. *Plant, Cell and Environment* **36:** 249-261.

Hatch MD. 1971. The C_4 pathway of photosynthesis. Evidence for an intermediate pool of carbon dioxide and the identity of the donor C_4-dicarboxylic acid. *Biochemical Journal* **125:** 425-432.

Hatterslay PW. 1984. Characterization of C_4 type leaf anatomy in grasses (Poaceae). Mesophyll:bundle sheath area ratios. *Annals of Botany* **53:** 163-180.

Ishikawa N, Takabayashi A, Sato F, Endo T. 2016. Accumulation of the components of cyclic electron flow around photosystem I in C_4 plants, with respect to the requirements for ATP. *Photosynthesis Research* **129:** 261-277.

Kramer DM, Evans JR. 2011. The importance of energy balance in improving photosynthetic productivity. *Plant Physiology* **155:** 70-78.

Majeran W, Cai Y, Sun Q, van Wijk LJ. 2005. Functional differentiation of bundle sheath and mesophyll maize chloroplasts determined by comparative proteomics. *The Plant Cell* **17:** 3111-3140.

Meister M, Agostino A, Hatch MD. 1996. The roles of malate and aspartate in C_4 photosynthetic metabolism of *Flaveria bidentis* (L). *Planta* **199:** 262-269.

Noctor G, Foyer CH. 1998. A re-evaluation of the ATP:NADPH budget during C_3 photosynthesis: a contribution from nitrate assimilation and its associated respiratory activity? *Journal of Experimental Botany* **49:** 1895-1908.

Takabayashi A, Kishine M, Asada K, Endo T, Sato F. 2005. Differential use of two cyclic electron flows around photosystem I for driving CO_2-concentration mechanism in C_4 photosynthesis. *Proceedings of the National Academy of Sciences of USA* **102:** 16898-16903.

van Kraalingen DWG, Rappoldt C., van Laar HH. 2003. The FORTRAN simulation translator, a simulation language. *European Journal of Agronomy* **18:** 259-361.

von Caemmerer S, Furbank RT. 1999. Modeling C_4 photosynthesis. In: Sage RF, Monson RK, eds. *C_4 Plant Biology*. Academic Press, Toronto, 173-211.

von Caemmerer S, Furbank RT. 2003. The C_4 pathway: an efficient CO_2 pump. *Photosynthesis Research* **77:** 191-207.

Wang Y, Bräutigam A, Weber APM, Zhu X-G. 2014. Three distinct biochemical subtypes of C_4 photosynthesis? A modelling analysis. *Journal of Experimental Botany* **65:** 3567-3578.

Yin X, Struik PC. 2012. Mathematical review of the energy transduction stoichiometries of C_4 leaf photosynthesis under limiting light. *Plant, Cell and Environment* **35:** 1299-1312.

Yin X, van Oijen M, Schapendonk AHCM. 2004. Extension of a biochemical model for the generalized stoichiometry of electron transport limited C_3 photosynthesis. *Plant, Cell and Environment* **27:** 1211-1222.
